# Supplementary material for: Raising Awareness of the Severity of “Contactless Stings” by Cassiopea Jellyfish and Kin
Source: Animals (Basel). 2021 Nov 24;11(12):3357. doi: 10.3390/ani11123357 (PMC8698115; doi:10.3390/ani11123357)
Supplement: Supplementary file 1 [file animals-11-03357-s001.zip › animals-1434736-supplementary/animals-1434736-Proofed Supplementary/animals-1434736-Supplementary Table S1.pdf]

Supplementary Table S1: Spearman's Rho

| Condition | *In situ* accounts | Aquarium system accounts | All Together |
|---|---|---|---|
| 2m radius medusa density | $R_s$=-0.219126, p-value = 0.3674 | $R_s$=*0.1768387, p-value = 0.4689* | $R_s$=0.1456426, p-value = 0.3829 |
| Medusa size | $R_s$=-0.1739114, p-value = 0.4764 | $R_s$=0.3681628, p-value = 0.1209 | $R_s$=0.1176921, p-value = 0.4942 |
| Distance from nearest medusa | $R_s$=0.2371207, p-value = 0.3283 | $R_s$=-0.1582722, p-value = 0.5175 | $R_s$=0.0589316, p-value = 0.7253 |
| Distance from highest medusa density | $R_s$=-0.2485889, p-value = 0.3716 | *NA (All less than 1m)* | $R_s$=-0.2162371, p-value = 0.2193 |
| Highest medusa density | $R_s$=-0.181472, p-value = 0.4572 | $R_s$=0.06200589, p-value = 0.8069 | $R_s$=0.1591033, p-value = 0.4942 |
| Time in water near medusae | $R_s$=-0.4919112, p-value = 0.06241 | $R_s$=0.04716038, p-value = 0.848 | $R_s$=-0.1072149, p-value = 0.5217 |

Spearman's rank coefficient calculated for correlation between each of the above features of an experience and self-reported pain level.
