# Supplementary material for: Raising Awareness of the Severity of “Contactless Stings” by Cassiopea Jellyfish and Kin
Source: Animals (Basel). 2021 Nov 24;11(12):3357. doi: 10.3390/ani11123357 (PMC8698115; doi:10.3390/ani11123357)
Supplement: Supplementary file 1 [file animals-11-03357-s001.zip › animals-1434736-supplementary/animals-1434736-Proofed Supplementary/Surveys/Stinging Water Survey (Bahasa).pdf]

Survey Tentang Air yang Menyengat

Air yang menyengat atau stinging water adalah sebuah sensasi ketidaknyamanan yang tidak diharapkan, yang dialami oleh para penikmat kegiatan air di daerah yang dipenuhi oleh Medusa dari species Cassiopea. Kasus air yang menyengat sangat banyak dijumpai, karena semakin banyak orang yang melaporkan kejadian ini bahkan ketika mereka sama sekali tidak bersentuhan dengan medusa ini. Secara umum, iritasi yang terjadi terbatas pada kulit bagian luar yang terendam di dalam air. Seperti yang dilaporkan dalam jurnal Nature Communications Biology (https://www.nature.com/articles/s42003-020-0777-8), Medusa dari Cassiopea menghasilkan mukus dengan jumlah "cassiosomes" yang sangat banyak. Cassiosomes merupakan bagian dari medusa yang memiliki kandungan nematocyst yang mengakibatkan rasa sakit mulai dari level yang rendah sampai dengan menyakitkan yang lebih dikenal dengan "stinging water" atau air yang menyengat.
Survey ini dilakukan oleh beberapa penulis dari jurnal tersebut diatas (Kaden Muffett, Anna Klompen, Cheryl Ames and Allen Collins) untuk menentukan situasi seperti apa yang mengakibatkan terjadinya stinging water, tempat terjadinya stingin water dan kisaran rasa sakit yang dialami ketika hal ini terjadi. Survey dilakukan dengan tujuan untuk mengumpulkan data yang akan dipublikasikan kedalam jurnal ilmiah. Para peserta survey setidaknya membutuhkan waktu 6-8 menit untuk melengkapi survey ini. Jika anda mengalami lebih dari satu kejadian, kami berharap anda menggunakan ketiga bagian dalam form ini dan melaporakan sampai dengan tiga kejadian. Walaupun kami tidak mengumpulkan dan mempublikasi informasi pribadi dalam survey ini, seperti umumumnya survei online, kami tidak memastikan kerahasiaan dari jawaban yang diberikan, mengingat pembuat survey memiliki akses ke jawaban yang anda berikan.

* Required

Judul Penelitian : Survey Interaksi Air yang Menyengat
Peneliti : Maria Pia Miglietta

Kenapa saya diharapakan untuk terlibat dalam penelitian ini?
Anda di harapakan untuk terlibat dalam survey ini karena kami berusaha untuk mempelajari pengalaman yang di alami oleh para peneliti dan aquarist berkaitan dengan phenomena stinging water ketika berada di sekitar rhizostomal jellyfish

Anda dipilih sebagai peserta atau partisipan dalam studi ini karena anda merespond permintaan kami untuk menjadi sukarelawan. Anda harus berusia 18 tahun atau lebih untuk dapat berpartispasi dalam penelitian ini.

Mengapa survey ini dilakukan?
Survey ini di desain untuk menentukan situasi seperti apa yang mengakibatkan terjadinya kejadian "Stinging Water", lokasi kejadian dan kisaran rasa sakit yang terjadi akibat kejadian tersebut. Survey ini di buat dengan tujuan untuk publikasi data ilmiah yang akan dimasukkan kedalam jurnal Communications Biology dalam bentuk "short invited communication"

Berapa lama survey ini akan berlangsung?
Survey ini akan berlangsung sekitar 7 sampai dengan 30 menit tergantung dari jumlah pengalaman yang ingin anda bagi dalam survey ini.

Lembar Persetujuan

Apa yang akan terjadi jika saya menyatakan bersedia untuk berpartispasi dalam survey ini?
Jika anda memilih untuk berpartisipasi, silahkan pilih “Saya Setuju” pada akhir dari bagian ini

Apa yang akan terjadi jika saya menyatakan tidak bersedia untuk berpartispasi dalam survey ini?
Partispasi anda dalam kegiatan ini bersifat sukarela. Anda dapat memilih untuk tidak berpartisipasi dan hal ini tidak akan berdampak terhadap kenyaman anda. Anda dapat meninggalkan survey ini kapanpun.

Apakah ada kemungkinan bahwa survey ini membahayakan saya?
Tidak ada pertanyaan yang sensitif dalam survey ini yang dapat mengakibatkan ketidaknyamanan. Bagaimanapun, anda dapat mengabaikan pertanyaan yang tidak ingin anda jawab. Anda juga dapat mengakhiri survey ini kapanpun jika anda menghendaki

Apa yang akan terjadi pada data dan informasi yang telah dikumpulkan?
Anda dapat mengakses peraturan tentang kerahasiaan dari survey ini di https://policies.google.com/privacy. Tidak ada informasi pribadi yang akan dikumpulkan, kecuali anda secara sukarela meminta untuk dimasukkan kedalam halaman acknowledgement dari paper yang akan diterbitkan

Informasi anda akan disimpan secara rahasia sesuai dengan hukum yang berlaku. Hasil dari survey ini akan diterbitkan dalam publikasi ilmiah dan kami akan memastikan informasi pribadi anda tetap rahasia.

Dengan siapa anda dapat berkomunikasi?
Jika anda memiliki pertanyaan seputar penelitian ini, anda dapat menghubung nomor +1 202-368-8338 dan atau bisa menulis surat elektronik ke kmmuffett@tamu.edu. Anda juga dapat menghubungi bagian Human Research Protection Program di Texas A&M University (sekelompok orang yang bertugas untuk melakukan review terhadap kegiatan penelitian dan melindungi hak orang-orang yang terlibat pada penelitian tersebut) di nomer 1-979-458-4067, atau telepon bebas biaya di 1-855-795-8636, atau menulis email ke irb@tamu.edu untuk:
• Meminta bantuan terkait pertanyaan-pertanyaan yang muncul tentang penelitian ini.
• Menyatakan keluhan dan keprihatinan terhadap penelitian ini
• Mendapatkan jawaban atas pertanyaan terkait hak sebagai peserta penelitian
• Mendapatkan tanggapan dari staff yang bertanggung jawab terhadap penelitian ini
• Berbicara dengan seseorang diluar staff yang bertanggung jawab terhadap penelitian ini

Jika anda mengharapakan salinan dari persetujuan ini, anda dapat melakukannya melalui menu “Print” yang tersedia di layar

☐ Jika anda ingin berpartispasi dalam survey ini, silahkan pilih “Saya Setuju” atau pilih X di ujung atas browser anda

☐ Jika anda tidak ingin berpartispasi dalam survey ini, silahkan pilih “Saya Tidak Setuju” atau pilih X di ujung atas browser anda

1. Apakah anda setuju untuk berpartisipasi dalam penelitian ini *

Mark only one oval.

- Saya Setuju
- Saya Tidak Setuju

Informasi Umum

Silahkan jawab beberapa pertanyaan berikut ini sehingga kami dapat mengkategorikan pengalaman anda dengan baik.

2. Berapa lama anda sudah bekerja dengan Cassiopea or other Rhizostome jellyfish?

Mark only one oval.

- Saya tidak pernah bekerja dengan jellyfish untuk keperluan riset atau tujuan aquarium
- <1 tahun
- 1-3 tahun
- 3-6 tahun
- 6+ tahun

3. Berapa kali anda mengalami kejadian “stinging water”?

Mark only one oval.

- Sekali
- Dua kali
- Tiga kali
- Lebih dari tiga kali
- Tidak pernah mengalami *Skip to question 59*

4. Saya mengalami kejadian stinging water ketika saya berperan sebagai?

Check all that apply.

- [ ] Professional aquarist
- [ ] Pemilik aquarium rumahan
- [ ] Peneliti (termasuk mahasiswa master atau doktoral)
- [ ] Mahasiswa (tidak termasuk mahasiswa master atau doktoral)
- [ ] Kegiatan rekreasi (berenang/snorkeling)

Other: [ ] ____________________

Kejadian 1

Mohon jelaskan hanya satu kejadian pada bagian ini

5. Sebutkan secara geographic (selengkap dan seakurat mungkin) tentang dimana kejadian ini terjadi ?

____

6. Jika anda mengetahui titik koordinat dari lokasi ini, silahakan isi di kolom dibawah ini. Jika tidak silahkan mengabaikan pertanyaan ini

____

7. Bagaimana anda mengklasifikasikan tempat tersebut?

Mark only one oval.

- Daerah Pohon Mangrove atau Bakau
- Lagoon
- Danau quarry atau danau dari bekas galian tambang
- Laut
- Fasiltas Aquarium di ruang Publik
- Fasilitas Aquarium di rumah
- Other: ____________________

8. Ketika anda mengalami kejadian ini, apa yang sedang anda lakukan?

Mark only one oval.

- Berjalan di pinggir atau disekitar pantai
- Menyelam dengan SCUBA
- Snorkling
- Bekerja di sekitar lingkungan aquarium
- Other: ____________________

9. Jika kejadian ini terjadi, seberapa dekat anda dengan medusa dari Cassiopea

Mark only one oval.

- <10 cm
- 10-50 cm
- 50-100 cm
- 1-2 m
- 2-5 m
- > 5 m
- Tidak tahu atau tidak melihat Cassiopea
- Tidak dekat dengan Cassiopea

10. Sama dengan pertanyaan diatas tetapi untuk jenis jellyfish yang lain. Berikan nama spesies di bagian selanjutnya jika anda yakin dengan nama spesies tersebut

Mark only one oval.

- <10 cm
- 10-50 cm
- 50-100 cm
- 1-2 m
- 2-5 m
- >5 m

11. Jika bukan dari jenis jellyfish Cassiopea, sebutkan nama genus atau spesies. Beberapa spesies rhizostome jellyfish umum yang dijumpai diberikan pada gambar diatas.

12. Berdasarkan observasi terbaik anda, berapa jumlah Cassiopea (atau spesies lain) yang ada disekitar radius 2 meter disekeliling anda?

Mark only one oval.

- 0
- 1
- 2-5
- 5-10
- 10-20
- 20+

13. Berkaitan pertanayaan diatas, berikan perkiraan ukuran rata-rata dari medusae di sekilling anda (dalam radius 2 m)

Mark only one oval.

- <5 cm
- 5-10 cm
- 10-15 cm
- 15-30 cm
- Other: ____________________

14. Berapa jumlah kepadatan terbanyak dari Cassiopea (atau dari jellyfish jenis lain) di daerah dimana anda pernah bekerja (jumlah individu/m2)

____

15. Seberapa jauh anda dari daerah dengan jumlah kepadatan tertinggi ini?

Mark only one oval.

- <1 m
- 1-2 m
- 2-5 m
- 5-8 m
- >8 m
- Tidak melihat atau tidak tahu
- Tidak ada Rhizostome jellyfish di sekitar anda

16. Apakah anda melakukan sesuatu yang mengakibatkan menyebarnya kerumunan dari medusa tersebut ? Kegiatan ini meliputi menyetuh medusae tersebut, menendang daerah disekitar anda dengan fins, menginjak medusae tersebut atau yang lainnya.

Mark only one oval.

- Ya
- Tidak
- Mungkin

17. Berapa lama anda berada di daerah tersebut

Mark only one oval.

- <5 min
- 5-10 min
- 10-15 min
- 15 -30 min
- 30-60 min
- 1 hr+

18. Sebutkan level ketidaknyamanan yang anda alami (1. Sentuhan ringan, 3. Iritasi dan rasa perih, 5. Rasa sakit yang amat sangat)

Mark only one oval.

	1	2	3	4	5	
Sentuhan ringan	⬭	⬭	⬭	⬭	⬭	Rasa sakit yang amat sangat

19. Jika anda harus membandingkan rasa sakit yang anda alami, rasa sakit jenis apa yang anda bisa bandingkan dengan kejadian diatas?

____

20. Apakah ada perubahan warna kulit di daerah yang mengalami kontak dengan jellyfish tersebut?

Mark only one oval.

- ⬭ Iya
- ⬭ Tidak
- ⬭ Saya tidak tahu

21. Apakah ada hal lain yang ingin anda ceritakan tentang kejadian atau pengalaman ini?

____
____
____
____
____

22. Apakah anda memiliki detail lain yang ingin anda ceritakan?

Mark only one oval.

- Ya (anda dapat mengulangi pertanyaan diatas untuk memeberikan penjelasan tambahan)
- Tidak (Informasi anda akan dimasukkan dan anda akan diminta untuk memberikan izin publikasi dari informasi yang sudah anda berikan) *Skip to question 59*

Kejadian 2

Mohon jelaskan hanya satu kejadian pada bagian ini

23. Sebutkan secara geographic (selengkap dan seakurat mungkin) tentang dimana kejadian ini terjadi ?

24. Jika anda mengetahui titik koordinat dari lokasi ini, silahakan isi di kolom dibawah ini. Jika tidak silahkan mengabaikan pertanyaan ini

25. Bagaimana anda mengklasifikasikan tempat tersebut?

Mark only one oval.

- Daerah Pohon Mangrove atau Bakau
- Lagoon
- Danau quarry atau danau dari bekas galian tambang
- Laut
- Fasiltas Aquarium di ruang Publik
- Fasilitas Aquarium di rumah
- Other: ______________________

26. Ketika anda mengalami kejadian ini, apa yang sedang anda lakukan?

Mark only one oval.

- Berjalan di pinggir atau disekitar pantai
- Menyelam dengan SCUBA
- Snorkling
- Bekerja di sekitar lingkungan aquarium
- Other: ______________________

27. Jika kejadian ini terjadi, seberapa dekat anda dengan medusa dari Cassiopea

Mark only one oval.

- <10 cm
- 10-50 cm
- 50-100 cm
- 1-2 m
- 2-5 m
- > 5 m
- Tidak tahu atau tidak melihat Cassiopea
- Tidak dekat dengan Cassiopea

28. Sama dengan pertanyaan diatas tetapi untuk jenis jellyfish yang lain. Berikan nama spesies di bagian selanjutnya jika anda yakin dengan nama spesies tersebut

Mark only one oval.

- <10 cm
- 10-50 cm
- 50-100 cm
- 1-2 m
- 2-5 m
- >5 m

29. Jika bukan dari jenis jellyfish Cassiopea, sebutkan nama genus atau spesies. Beberapa spesies rhizostome jellyfish umum yang dijumpai diberikan pada gambar diatas.

____

30. Berdasarkan observasi terbaik anda, berapa jumlah Cassiopea (atau spesies lain) yang ada disekitar radius 2 meter disekeliling anda?

Mark only one oval.

- 0
- 1
- 2-5
- 5-10
- 10-20
- 20+

31. Berkaitan pertanayaan diatas, berikan perkiraan ukuran rata-rata dari medusae di sekilling anda (dalam radius 2 m)

Mark only one oval.

- <5 cm
- 5-10 cm
- 10-15 cm
- 15-30 cm
- Other: ____________________

32. Berapa jumlah kepadatan terbanyak dari Cassiopea (atau dari jellyfish jenis lain) di daerah dimana anda pernah bekerja (jumlah individu/m2)

____

33. Seberapa jauh anda dari daerah dengan jumlah kepadatan tertinggi ini?

Mark only one oval.

- <1 m
- 1-2 m
- 2-5 m
- 5-8 m
- >8 m
- Tidak melihat atau tidak tahu
- Tidak ada Rhizostome jellyfish di sekitar anda

34. Apakah anda melakukan sesuatu yang mengakibatkan menyebarnya kerumunan dari medusa tersebut ? Kegiatan ini meliputi menyetuh medusae tersebut, menendang daerah disekitar anda dengan fins, menginjak medusae tersebut atau yang lainnya.

Mark only one oval.

- Ya
- Tidak
- Mungkin

35. Berapa lama anda berada di daerah tersebut

Mark only one oval.

- <5 min
- 5-10 min
- 10-15 min
- 15 -30 min
- 30-60 min
- 1 hr+

36. Sebutkan level ketidaknyamanan yang anda alami (1. Sentuhan ringan, 3. Iritasi dan rasa perih, 5. Rasa sakit yang amat sangat)

Mark only one oval.

	1	2	3	4	5	
Sentuhan ringan	◯	◯	◯	◯	◯	Rasa sakit yang amat sangat

37. Jika anda harus membandingkan rasa sakit yang anda alami, rasa sakit jenis apa yang anda bisa bandingkan dengan kejadian diatas?

____

38. Apakah ada perubahan warna kulit di daerah yang mengalami kontak dengan jellyfish tersebut?

Mark only one oval.

◯ Iya
◯ Tidak
◯ Saya tidak tahu

39. Apakah ada hal lain yang ingin anda ceritakan tentang kejadian atau pengalaman ini?

____
____
____
____
____

40. Apakah anda memiliki detail lain yang ingin anda ceritakan?

Mark only one oval.

- Ya (anda dapat mengulangi pertanyaan diatas untuk memeberikan penjelasan tambahan)
- Tidak (Informasi anda akan dimasukkan dan anda akan diminta untuk memberikan izin publikasi dari informasi yang sudah anda berikan) *Skip to question 59*

Kejadian 3

Mohon jelaskan hanya satu kejadian pada bagian ini

41. Sebutkan secara geographic (selengkap dan seakurat mungkin) tentang dimana kejadian ini terjadi ?

42. Jika anda mengetahui titik koordinat dari lokasi ini, silahakan isi di kolom dibawah ini. Jika tidak silahkan mengabaikan pertanyaan ini

43. Bagaimana anda mengklasifikasikan tempat tersebut?

Mark only one oval.

- Daerah Pohon Mangrove atau Bakau
- Lagoon
- Danau quarry atau danau dari bekas galian tambang
- Laut
- Fasiltas Aquarium di ruang Publik
- Fasilitas Aquarium di rumah
- Other: ____________________

44. Ketika anda mengalami kejadian ini, apa yang sedang anda lakukan?

Mark only one oval.

- Berjalan di pinggir atau disekitar pantai
- Menyelam dengan SCUBA
- Snorkling
- Bekerja di sekitar lingkungan aquarium
- Other: ____________________

45. Jika kejadian ini terjadi, seberapa dekat anda dengan medusa dari Cassiopea

Mark only one oval.

- <10 cm
- 10-50 cm
- 50-100 cm
- 1-2 m
- 2-5 m
- > 5 m
- Tidak tahu atau tidak melihat Cassiopea
- Tidak dekat dengan Cassiopea

46. Sama dengan pertanyaan diatas tetapi untuk jenis jellyfish yang lain. Berikan nama spesies di bagian selanjutnya jika anda yakin dengan nama spesies tersebut

Mark only one oval.

- <10 cm
- 10-50 cm
- 50-100 cm
- 1-2 m
- 2-5 m
- >5 m

47. Jika bukan dari jenis jellyfish Cassiopea, sebutkan nama genus atau spesies. Beberapa spesies rhizostome jellyfish umum yang dijumpai diberikan pada gambar diatas.

____

48. Berdasarkan observasi terbaik anda, berapa jumlah Cassiopea (atau spesies lain) yang ada disekitar radius 2 meter disekeliling anda?

Mark only one oval.

- 0
- 1
- 2-5
- 5-10
- 10-20
- 20+

49. Berkaitan pertanayaan diatas, berikan perkiraan ukuran rata-rata dari medusae di sekilling anda (dalam radius 2 m)

Mark only one oval.

- <5 cm
- 5-10 cm
- 10-15 cm
- 15-30 cm
- Other: ______________________

50. Berapa jumlah kepadatan terbanyak dari Cassiopea (atau dari jellyfish jenis lain) di daerah dimana anda pernah bekerja (jumlah individu/m2)

____

51. Seberapa jauh anda dari daerah dengan jumlah kepadatan tertinggi ini?

Mark only one oval.

- <1 m
- 1-2 m
- 2-5 m
- 5-8 m
- >8 m
- Tidak melihat atau tidak tahu
- Tidak ada Rhizostome jellyfish di sekitar anda

52. Apakah anda melakukan sesuatu yang mengakibatkan menyebarnya kerumunan dari medusa tersebut ? Kegiatan ini meliputi menyetuh medusae tersebut, menendang daerah disekitar anda dengan fins, menginjak medusae tersebut atau yang lainnya.

Mark only one oval.

- Ya
- Tidak
- Mungkin

53. Berapa lama anda berada di daerah tersebut

Mark only one oval.

- <5 min
- 5-10 min
- 10-15 min
- 15 -30 min
- 30-60 min
- 1 hr+

54. Sebutkan level ketidaknyamanan yang anda alami (1. Sentuhan ringan, 3. Iritasi dan rasa perih, 5. Rasa sakit yang amat sangat)

Mark only one oval.

	1	2	3	4	5	
Sentuhan ringan	⬭	⬭	⬭	⬭	⬭	Rasa sakit yang amat sangat

55. Jika anda harus membandingkan rasa sakit yang anda alami, rasa sakit jenis apa yang anda bisa bandingkan dengan kejadian diatas?

56. Apakah ada perubahan warna kulit di daerah yang mengalami kontak dengan jellyfish tersebut?

Mark only one oval.

⬭ Iya

⬭ Tidak

⬭ Saya tidak tahu

57. Apakah ada hal lain yang ingin anda ceritakan tentang kejadian atau pengalaman ini?

58. Apakah anda memiliki detail lain yang ingin anda ceritakan?

Mark only one oval.

- Ya (anda dapat mengulangi pertanyaan diatas untuk memeberikan penjelasan tambahan)
- Tidak (Informasi anda akan dimasukkan dan anda akan diminta untuk memberikan izin publikasi dari informasi yang sudah anda berikan) *Skip to question 59*

Izin Penggunaan Data

Terima kasih atas informasi yang anda sudah berikan. Sebelum informasi ini anda serahkan, silahkan pilih beberapa pilihan dibawah ini untuk menyelesaikan penyerahan informasi yang anda berikan

59. Pemberian izin: Apakah anda memberikan izin dari informasi yang anda berikan untuk dipublikasikan dalam jurnal ilmiah? *

Mark only one oval.

- Tidak
- Ya, Anda dapat menggunakan informasi ini sebagai data poin
- Ya, Anda dapat menggunakan data dan informasi tertulis ini sebagai data poin
- Ya, Anda dapat menggunakan data dan informasi tertulis ini sebagai data poin dan mohon masukkan nama saya sebagai bagian dari halaman pengakuan atau acknowledgement

60. Nama untuk halaman pengakuan atau acknowledgment. Terima kasih atas informasi yang anda sudah berikan.

This content is neither created nor endorsed by Google.

Google Forms
