# Supplementary material for: Raising Awareness of the Severity of “Contactless Stings” by Cassiopea Jellyfish and Kin
Source: Animals (Basel). 2021 Nov 24;11(12):3357. doi: 10.3390/ani11123357 (PMC8698115; doi:10.3390/ani11123357)
Supplement: Supplementary file 1 [file animals-11-03357-s001.zip › animals-1434736-supplementary/animals-1434736-Proofed Supplementary/Surveys/Stinging Water Survey (Chinese).pdf]

"刺痛的水" 调查问卷

"刺痛的水"是浮潜者和跋涉者在含有仙女水母（Cassiopea medusae）的地区遇到意外不适的感觉。值得注意的是人们经常报告根本没有接触水母。通常，这种刺激仅发生在浸入水中的裸露皮肤。正如最近发表在《自然通讯生物学》（https://www.nature.com/articles/s42003-020-0777-8）上的一篇论文所报道的那样，仙女水母（Cassiopea medusae）产生的粘液含有大量的含有刺细胞团的结构，称为" cassiosomes"。导致令人不舒服的刺痛，这种现象称为"刺痛的水"。这项调查由本文的一些作者（Kaden Muffett，Anna Klompen，Cheryl Ames和Allen Collins）进行，以确定什么情况会导致这种"刺痛的水"的真实体验以及所导致的物理反应。此调查问卷的目的是计划要在科学期刊上发表。报告个人经历大约需要6-8分钟。如果您有多个经验，我们建议您使用此表单中的所有三个条目部分，并最多报告三种经验。虽然我们没有收集或发布个人信息，就像任何在线调查一样，我们不能保证您的答案的完全隐私，因为这个问卷调查的主办人保留了一些访问权限。

* Required

知情同意书

研究标题：刺痛的水的影响调查
调查人员：Maria Pia Miglietta

为什么要求我参加这项研究?
之所以邀请您参加此研究是因为我们正在尝试更多地了解研究人员和水族养殖者对根状水母周围"刺痛的水"现象的经验。
您被选为该研究的可能参与者，因为您响应了我们对志愿者的电子邮件请求。您必须年满18周岁才能参加。

为什么要进行这项研究?
该调查旨在确定哪些情况会导致"刺痛的水"的真实体验以及由此产生的物理反应范围。创建此调查的明确意图是在以在《自然通讯生物学》"（https://www.nature.com/articles/s42003-020-0777-8）中就该主题进行发布的邀请函。

完成这个问卷需要多长时间?
您将花费大约7到30分钟的时间，而且具体取决于您希望记录多少经验。

如果我说"是的，我想参与这项研究"会怎样?
如果您决定参加，请在本节末尾选择"我同意"。

如果我不想参加这项研究会怎样?
您的参与对于本研究是自愿的。您可以决定不参加此研究，并且不会对您不利。您可以随时离开调查。

这项研究有什么方法可以伤害我吗?
此调查中没有敏感问题会引起不适。但是，您可以跳过任何您不想回答的问题，也可以随时退出调查。

为了此研究收集的信息会怎样?
您可以通过以下网址查看调查主持人的保密政策：https：//policies.google.com/privacy
除非您自愿要求将其包含在确认中，否则不会收集任何直接的个人标识符。

您的信息将在法律允许的范围内保密。研究结果可能会公开，但您的身份将保密。

如果有问题的话我可以和谁联系?
请随时提出有关这项研究的问题。如果您还有其他疑问或疑虑，请致电202-368-8338或通过邮件kmmuffett@tamu.edu与Kade Muffett联系。

您还可以通过手机拨打1-979-458-4067与免费电话1-855-795-8636联系或通过电子邮件irb@tamu.edu发送德克萨斯A＆M大学的人类研究保护计划（该小组是一组审查研究以保护您的权利的人）关于发送以下信息：
•有关研究的任何问题的其他帮助。
•对研究表达疑虑或抱怨。
•获得有关您作为研究参与者的权利的问题的答案。
•关于您无法与研究人员交流的顾虑。
•与研究人员以外的人交谈的希望。

如果您需要此同意书的副本作为记录，可以从屏幕上打印它。
如果您想参加，请单击“我同意”按钮，您将被带到调查中。

如果您不希望参加本研究，请选择“我不同意”或在浏览器的一角选择退出。

1. 请问您同意参与此研究吗 *

Mark only one oval.

- 我同意
- 我不同意

基本信息

请提供一些信息以助于我们更好地对于您的经验进行分类。

2. 请问您一共与仙女水母或别的根状水母从事多久的工作呢?

Mark only one oval.

- 我从未将这些水母用于研究或水族馆用途
- < 1 年
- 1-3 年
- 3-6 年
- 6+ 年

3. 您有多少次感觉到“刺痛的水”？

Mark only one oval.

- 一次
- 两次
- 三次
- 大于三次
- 从未有过 *Skip to question 58*

4. 我经历了刺痛的水当我在作为以下角色时：

Check all that apply.

- [ ] 专业水族管理员
- [ ] 水族馆主
- [ ] 研究员（包括研究生）
- [ ] 学生（未毕业）
- [ ] 休闲游泳者 / 浮潜者， 在休闲活动中

Other: [ ] ____________________

经历1

请在这里仅详细介绍一次体验。

5. 请问可以提供具体地理位置（越具体越好）关于您经历过“刺痛的水”吗？

____

6. 如果您知道此位置的具体坐标， 请提供相关信息，否则请跳过这个问题。

____

7. 您愿意将此地点分类为：

Mark only one oval.

- 红树林
- 泻湖 / 咸水湖
- 采石场
- 海
- 公共水族馆（工作用）
- 私人水族馆（家中）
- Other: ____________

8. 当您感觉到这一点时，您在做什么（涉水，潜水，浮潜）？

Mark only one oval.

- 涉水 / 蹚水
- 潜水
- 浮潜
- 在水族馆工作时
- Other: ____________

9. 如果您接近过仙女水母，那么您离仙女水母有多近呢？

Mark only one oval.

- <10 cm
- 10-50 cm
- 50-100 cm
- 1-2 m
- 2-5 m
- > 5 m
- 没有看到或不清楚
- 并没有接近过仙女水母

10. 与上一个问题相同， 如果您遇见了其他种类的水母请选择您与其的距离并在下一个问题中提供物种的名字：

Mark only one oval.

- <10 cm
- 10-50 cm
- 50-100 cm
- 1-2 m
- 2-5 m
- >5 m

11. 如果不是仙女水母，请提供属名或种名。 上图中提供了一些常见的根状水母。

12. 据您所知，在您2m的半径内有多少只仙女水母（或其他目标根状水母）？

Mark only one oval.

- 0
- 1
- 2-5
- 5-10
- 10-20
- 20+

13. 提供在您附近附近水母的平均距离。 （半径2 m）

Mark only one oval.

- <5 cm
- 5-10 cm
- 10-15 cm
- 15-30 cm
- Other: ______________

14. 您在工作区域看到的最高的仙女水母（或其他目标根状水母）密度是多少（个数 / 平方米）？

____

15. 您距离这个密度最高的区域有多远?

Mark only one oval.

- <1 m
- 1-2 m
- 2-5 m
- 5-8 m
- >8 m
- 没有看到任何或不知道
- 在这个地区没有任何根状水母

16. 您是否进行过导致水母混乱或骚乱的动作？ 这包括戳动，用脚踢周围的水，踩水等等。

Mark only one oval.

- 有过
- 没有
- 也许有

17. 你在这个区域待了多久?

Mark only one oval.

- <5 分钟
- 5-10 分钟
- 10-15 分钟
- 15 -30 分钟
- 30-60 分钟
- 大于一小时

18. 请问您经历过的不适水平是？ （1-轻微的瘙痒，3-灼热和刺激，5-严重的疼痛）

Mark only one oval.

	1	2	3	4	5	
轻微的瘙痒	◯	◯	◯	◯	◯	严重的疼痛

19. 您认为这种不适最能比作什么？

____

20. 在经历这些后您有没有任何皮肤变色的经历呢？

Mark only one oval.

◯ 有
◯ 没有
◯ 我不知道

21. 您还有其他要告诉我们的情况吗？

____
____
____
____
____

22. 您还有其他想详细说明的经历吗？

Mark only one oval.

◯ 是（单击“是”将使您重复这些问题以获得您的另一个经历）
◯ 否（单击否将使您进入使用许可和最终提交） *Skip to question 58*

经历2

请在这里仅详细介绍一次体验。

23. 请问可以提供具体地理位置（越具体越好）关于您经历过“刺痛的水”吗？

24. 如果您知道此位置的具体坐标， 请提供相关信息，否则请跳过这个问题。

25. 您愿意将此地点分类为：

Mark only one oval.

- 红树林
- 泻湖 / 咸水湖
- 采石场
- 海
- 公共水族馆（工作用）
- 私人水族馆
- Other: ____________

26. 当您感觉到这一点时，您在做什么（涉水，潜水，浮潜）？

Mark only one oval.

- ⬭ 涉水 / 蹚水
- ⬭ 潜水
- ⬭ 浮潜
- ⬭ 在水族馆工作时
- ⬭ Other: ____________

27. 如果您接近过仙女水母，那么您离仙女水母有多近呢?

Mark only one oval.

- ⬭ <10 cm
- ⬭ 10-50 cm
- ⬭ 50-100 cm
- ⬭ 1-2 m
- ⬭ 2-5 m
- ⬭ > 5 m
- ⬭ 没有看到或不清楚
- ⬭ 并没有接近过仙女水母

28. 与上一个问题相同， 如果您遇见了其他种类的水母请选择您与其的距离并在下一个问题中提供物种的名字：

Mark only one oval.

- <10 cm
- 10-50 cm
- 50-100 cm
- 1-2 m
- 2-5 m
- >5 m

29. 如果不是仙女水母，请提供属名或种名。 上图中提供了一些常见的根状水母。

30. 据您所知，在您2m的半径内有多少只仙女水母（或其他目标根状水母）？

Mark only one oval.

- 0
- 1
- 2-5
- 5-10
- 10-20
- 20+

31. 提供在您附近附近水母的平均距离。（半径2 m）

Mark only one oval.

- <5 cm
- 5-10 cm
- 10-15 cm
- 15-30 cm
- Other: ______________________

32. 您在工作区域看到的最高的仙女水母（或其他目标根状水母）密度是多少（个数 / 平方米）？

____

33. 您距离这个密度最高的区域有多远?

Mark only one oval.

- <1 m
- 1-2 m
- 2-5 m
- 5-8 m
- >8 m
- 没有看到任何或不知道
- 在这个地区没有任何根状水母

34. 您是否进行过导致水母混乱或骚乱的动作？ 这包括戳动，用脚踢周围的水，踩水等等。

Mark only one oval.

- 有过
- 没有
- 也许有

35. 你在这个区域待了多久?

Mark only one oval.

- <5 分钟
- 5-10 分钟
- 10-15 分钟
- 15 -30 分钟
- 30-60 分钟
- 大于一小时

36. 请问您经历过的不适水平是？ （1-轻微的瘙痒，3-灼热和刺激，5-严重的疼痛）

Mark only one oval.

	1	2	3	4	5	
轻微的瘙痒						严重的疼痛

37. 您认为这种不适最能比作什么?

38. 在经历这些后您有没有任何皮肤变色的经历呢?

Mark only one oval.

- 有
- 没有
- 我不知道

39. 您还有其他要告诉我们的情况吗?

40. 您还有其他想详细说明的经历吗?

Mark only one oval.

- 是（单击“是”将使您重复这些问题以获得您的另一个经历）
- 否（单击否将使您进入使用许可和最终提交） *Skip to question 58*

经历3

请在这里仅详细介绍一次体验。

41. 请问可以提供具体地理位置（越具体越好）关于您经历过“刺痛的水”吗?

42. 如果您知道此位置的具体坐标， 请提供相关信息，否则请跳过这个问题。

____

43. 您愿意将此地点分类为：

Mark only one oval.

- ◯ 红树林
- ◯ 泻湖 / 咸水湖
- ◯ 采石场
- ◯ 海
- ◯ 公共水族馆（工作用）
- ◯ 私人水族馆（家中）
- ◯ Other: ______________________________

44. 当您感觉到这一点时，您在做什么（涉水，潜水，浮潜）？

Mark only one oval.

- ◯ 涉水 / 蹚水
- ◯ 潜水
- ◯ 浮潜
- ◯ 在水族馆工作时
- ◯ Other: ______________________________

45. 如果您接近过仙女水母，那么您离仙女水母有多近呢？

Mark only one oval.

- <10 cm
- 10-50 cm
- 50-100 cm
- 1-2 m
- 2-5 m
- > 5 m
- 没有看到或不清楚
- 并没有接近过仙女水母

46. 与上一个问题相同， 如果您遇见了其他种类的水母请选择您与其的距离并在下一个问题中提供物种的名字：

Mark only one oval.

- <10 cm
- 10-50 cm
- 50-100 cm
- 1-2 m
- 2-5 m
- >5 m

47. 如果不是仙女水母，请提供属名或种名。 上图中提供了一些常见的根状水母。

____

48. 据您所知，在您2m的半径内有多少只仙女水母（或其他目标根状水母）？

Mark only one oval.

- 0
- 1
- 2-5
- 5-10
- 10-20
- 20+

49. 提供在您附近附近水母的平均距离。（半径2 m）

Mark only one oval.

- <5 cm
- 5-10 cm
- 10-15 cm
- 15-30 cm
- Other: ____________

50. 您在工作区域看到的最高的仙女水母（或其他目标根状水母）密度是多少（个数 / 平方米）？

____

51. 您距离这个密度最高的区域有多远?

Mark only one oval.

- <1 m
- 1-2 m
- 2-5 m
- 5-8 m
- >8 m
- 没有看到任何或不知道
- 在这个地区没有任何根状水母

52. 您是否进行过导致水母混乱或骚乱的动作？ 这包括戳动，用脚踢周围的水，踩水等等。

Mark only one oval.

- 有过
- 没有
- 也许有

53. 你在这个区域待了多久?

Mark only one oval.

- <5 分钟
- 5-10 分钟
- 10-15 分钟
- 15 -30 分钟
- 30-60 分钟
- 大于一小时

54. 请问您经历过的不适水平是？ （1-轻微的瘙痒，3-灼热和刺激，5-严重的疼痛）

Mark only one oval.

	1	2	3	4	5	
轻微的瘙痒	⬭	⬭	⬭	⬭	⬭	严重的疼痛

55. 您认为这种不适最能比作什么？

____

56. 在经历这些后您有没有任何皮肤变色的经历呢？

Mark only one oval.

- ⬭ 有
- ⬭ 没有
- ⬭ 我不知道

57. 您还有其他要告诉我们的情况吗？

____

____

____

____

____

使用许可

谢谢你的回复。 提交之前，请选择以下选项以发布此信息。

58. 许可：您是否同意在公共期刊交流中使用有关非接触式水母刺伤经历的调查答案？ *

Mark only one oval.

- 不同意
- 是的，您可以将我的答案用作数据。
- 是的，您可以将我的答案用作数据和匿名的书面答复。
- 是的，您可以将我的答案用作数据和书面答复，但请在感谢栏中分别提供我的名字。

59. 请提供您的名字以至于我们列在感谢栏：感谢您的回复

This content is neither created nor endorsed by Google.

Google Forms
