# Supplementary material for: Raising Awareness of the Severity of “Contactless Stings” by Cassiopea Jellyfish and Kin
Source: Animals (Basel). 2021 Nov 24;11(12):3357. doi: 10.3390/ani11123357 (PMC8698115; doi:10.3390/ani11123357)
Supplement: Supplementary file 1 [file animals-11-03357-s001.zip › animals-1434736-supplementary/animals-1434736-Proofed Supplementary/Surveys/Stinging Water Survey (English).pdf]

# Stinging Water Survey

"Stinging water" is the sensation of unexpected discomfort experienced by snorkelers and waders in areas containing Cassiopea medusae. This is notable as people often report not touching the medusae at all. Generally, the irritation is restricted to exposed skin that is submerged. As reported in a recent paper in Nature Communications Biology ( https://www.nature.com/articles/s42003-020-0777-8 ), Cassiopea medusae produce mucus with a high load of nematocyst-containing structures known as "cassiosomes" that results in an uncomfortable to painful sting known as "stinging water". This survey is being conducted by some of the authors of this paper (Kaden Muffett, Anna Klompen, Cheryl Ames and Allen Collins) to determine what situations lead to real-world experiences of this "Stinging Water" and the range of physical reactions resulting. This survey is being distributed with express intent for publication in a scientific journal. Reporting an individual experience should take roughly 6-8 minutes. If you have more than one experience, we encourage you to use all three entry sections within this form and report up to three experiences. While we are not collecting or publishing personal information, as with any online survey, we cannot guarantee the complete privacy of your answers, as the survey host retains some access privileges.

* Required

## Informed Consent

Title of Research Study: Stinging Water Interactions Survey
Investigator: Maria Pia Miglietta

Why am I being asked to take part in this research study?
You are invited to participate in this study because we are trying to learn more about researchers' and aquarists' experiences with the stinging water phenomenon around rhizostomal jellyfish.

You were selected as a possible participant in this study because you responded to our email request for volunteers. You must be 18 years of age or older to participate.

Why is this research being done?
The survey is designed to determine what situations lead to real-world experiences of "Stinging Water" and the range of physical reactions resulting. This survey is being created with the express intent of publishing a short invited communication in Communications Biology on this topic.

How long is the survey?
It will take about 7 to 30 minutes of your time depending on how many experiences you wish to record.

What happens if I say "Yes, I want to be in this research"?
If you decide to participate, please select "I Agree" at the end of this section.

What happens if I do not want to be in this research?
Your participation in this study is voluntary. You can decide not to participate in this research and it will not be held against you. You can leave the survey at any time.

Is there any way being in this study could harm me?
There are no sensitive questions in this survey that should cause discomfort. However, you can skip any question you do not wish to answer, or exit the survey at any point.

What happens to the information collected for the research?
You may view the survey host's confidentiality policy at: https://policies.google.com/privacy
No direct personal identifiers will be collected UNLESS you voluntarily ask to be included in the acknowledgments.

Your information will be kept confidential to the extent allowed by law. The results of the research study may be published but your identity will remain confidential.

Who can I talk to?
Please feel free to ask questions regarding this study. You may contact Kade Muffett now or later if you have additional questions or concerns at 202-368-8338 and kmmuffett@tamu.edu.
You may also contact the Human Research Protection Program at Texas A&M University (which is a group of people who review the research to protect your rights) by phone at 1-979-458-4067, toll free at 1-855-795-8636, or by email at irb@tamu.edu for:

- additional help with any questions about the research
- voicing concerns or complaints about the research
- obtaining answers to questions about your rights as a research participant
- concerns in the event the research staff could not be reached
- the desire to talk to someone other than the research staff

If you want a copy of this consent for your records, you can print it from the screen.
If you wish to participate, please click the "I Agree" button and you will be taken to the survey.

If you do not wish to participate in this study, please select "I Disagree" or select X in the corner of your browser

1. Do you agree to participate in this study? *

*Mark only one oval.*

- I Agree
- I Disagree

## General Information

Please provide some information so that we can better categorize your experiences.

2. How long (total) have you worked with Cassiopea or other Rhizostome jellyfish?

*Mark only one oval.*

- I have not worked with these jellyfish for research or aquarium purposes
- < 1 year
- 1-3 years
- 3-6 years
- 6+ years

3. How many times have you felt "stinging water"?

*Mark only one oval.*

- Once
- Twice
- Thrice
- More than thrice
- Never *Skip to question 58*

4. I experienced stinging water while acting in my role as:

*Check all that apply.*

- [ ] Professional aquarist
- [ ] Home aquarium owner
- [ ] Researcher (including graduate students)
- [ ] Student (non-graduate)
- [ ] Recreational swimmer/snorkeler, during other leisure activities

Other: [ ] ______________________

## Experience 1

Please detail only a single experience here.

5. Where geographically (as specific as possible) did you feel “stinging water”?

______________________________________________

______________________________________________

______________________________________________

______________________________________________

______________________________________________

6. Where geographically (as specific as possible) did you feel “stinging water”?

______________________

7. What would you classify this location as

*Mark only one oval.*

- Mangrove
- Lagoon
- Quarry
- Sea
- Public Aquarium (i.e. work)
- Private Aquarium (i.e. home)
- Other: ______________________

8. When you felt this, what were you doing (wading, scuba, snorkel)?

*Mark only one oval.*

- Wading
- Scuba diving
- Snorkeling
- Working in an aquarium setting
- Other: ______________________

9. If you were close to a Cassiopea, how close to Cassiopea medusae were you?

*Mark only one oval.*

- <10 cm
- 10-50 cm
- 50-100 cm
- 1-2 m
- 2-5 m
- > 5 m
- Didn't see any or do not know
- Wasn't close to a Cassiopea

10. Same as above but for any kind of jellyfish. Provide species in next question if certain.

*Mark only one oval.*

- <10 cm
- 10-50 cm
- 50-100 cm
- 1-2 m
- 2-5 m
- >5 m

11. If non-Cassiopea jellyfish, provide genus or species name. Some common rhizostome jellyfish are provided in the images above.

12. To the best of your knowledge, how many Cassiopea (or other target Rhizostome jellyfish) were there in a 2 m radius of you?

*Mark only one oval.*

- 0
- 1
- 2-5
- 5-10
- 10-20
- 20+

13. Provide an average size estimate of medusae in your immediate vicinity. (2 m radius)

*Mark only one oval.*

- <5 cm
- 5-10 cm
- 10-15 cm
- 15-30 cm
- Other: ____________________

14. What was the highest Cassiopea (or other target Rhizostome jellyfish) density you saw in the area in which you were working (individuals/sq. meter)?

____________________

15. How far were you from this area of highest density?

*Mark only one oval.*

- <1 m
- 1-2 m
- 2-5 m
- 5-8 m
- >8 m
- Didn't see any or do not know
- No rhizostome jellyfish in the area

16. Did you engage in any action that resulted in disturbance or dislocation of medusae? This includes poking, kicking up surrounding water with fins, stepping on, etc.

*Mark only one oval.*

- Yes
- No
- Maybe

17. How long did you stay in this area?

*Mark only one oval.*

- <5 min
- 5-10 min
- 10-15 min
- 15 -30 min
- 30-60 min
- 1 hr+

18. What level of discomfort did you experience? (1- mild tickle, 3- burn and irritation, 5- severe pain)

*Mark only one oval.*

| | 1 | 2 | 3 | 4 | 5 | |
|---|---|---|---|---|---|---|
| Mild tickle | ⬭ | ⬭ | ⬭ | ⬭ | ⬭ | Severe pain |

19. What would you consider this discomfort most comparable to?

____________________________

20. Did you experience any skin discoloration in this area post-discomfort?

*Mark only one oval.*

- ⬭ Yes
- ⬭ No
- ⬭ Don't know

21. Is there anything else you would like to tell us about this instance?

________________________________________________________

________________________________________________________

________________________________________________________

________________________________________________________

________________________________________________________

22. Do you have another experience you wish to detail?

*Mark only one oval.*

- ( ) Yes (Clicking yes will allow you to repeat these questions for an additional experience)
- ( ) No (Clicking no will send you to the Permissions for Use and final submission) *Skip to question 58*

## Experience 2

Please detail only a single experience here.

23. Where geographically (as specific as possible) did you feel "stinging water"?

_______________________________________________
_______________________________________________
_______________________________________________
_______________________________________________
_______________________________________________

24. If you know the coordinates of this location, please include them below, if not skip this question.

_______________________

25. What would you classify this location as

*Mark only one oval.*

- Mangrove
- Lagoon
- Quarry
- Sea
- Public Aquarium (i.e. work)
- Private Aquarium (i.e. home)
- Other: ______________________

26. When you felt this, what were you doing (wading, scuba, snorkel)?

*Mark only one oval.*

- Wading
- Scuba diving
- Snorkeling
- Working in an aquarium setting
- Other: ______________________

27. If you were close to a Cassiopea, how close to Cassiopea medusae were you?

*Mark only one oval.*

- <10 cm
- 10-50 cm
- 50-100 cm
- 1-2 m
- 2-5 m
- > 5 m
- Didn't see any or do not know
- Wasn't close to a Cassiopea

28. Same as above but for any kind of jellyfish. Provide species in next question if certain.

*Mark only one oval.*

- <10 cm
- 10-50 cm
- 50-100 cm
- 1-2 m
- 2-5 m
- >5 m

29. If non-Cassiopea jellyfish, provide genus or species name. Some common rhizostome jellyfish are provided in the images above.

______________________________

30. To the best of your knowledge, how many Cassiopea (or other target Rhizostome jellyfish) were there in a 2 m radius of you?

*Mark only one oval.*

- 0
- 1
- 2-5
- 5-10
- 10-20
- 20+

31. Provide an average size estimate of medusae in your immediate vicinity. (2 m radius)

*Mark only one oval.*

- <5 cm
- 5-10 cm
- 10-15 cm
- 15-30 cm
- Other: ______________________

32. What was the highest Cassiopea (or other target Rhizostome jellyfish) density you saw in the area in which you were working (individuals/sq. meter)?

______________________

33. How far were you from this area of highest density?

*Mark only one oval.*

- <1 m
- 1-2 m
- 2-5 m
- 5-8 m
- >8 m
- Didn't see any or do not know
- No rhizostome jellyfish in the area

34. Did you engage in any action that resulted in disturbance or dislocation of medusae? This includes poking, kicking up surrounding water with fins, stepping on, etc.

*Mark only one oval.*

- Yes
- No
- Maybe

35. How long did you stay in this area?

*Mark only one oval.*

- <5 min
- 5-10 min
- 10-15 min
- 15 -30 min
- 30-60 min
- 1 hr+

36. What level of discomfort did you experience? (1- mild tickle, 3- burn and irritation, 5- severe pain)

*Mark only one oval.*

| | 1 | 2 | 3 | 4 | 5 | |
|---|---|---|---|---|---|---|
| Mild tickle | ⬭ | ⬭ | ⬭ | ⬭ | ⬭ | Severe pain |

37. What would you consider this discomfort most comparable to?

______________________________

38. Did you experience any skin discoloration in this area post-discomfort?

*Mark only one oval.*

- Yes
- No
- Don't know

39. Is there anything else you would like to tell us about this instance?

40. Do you have another experience you wish to detail?

*Mark only one oval.*

- Yes (Clicking yes will allow you to repeat these questions for an additional experience)
- No (Clicking no will send you to the Permissions for Use and final submission) *Skip to question 58*

Experience 3

Please detail only a single experience here.

41. Where geographically (as specific as possible) did you feel "stinging water"?

42. If you know the coordinates of this location, please include them below, if not skip this question.

43. What would you classify this location as

*Mark only one oval.*

- Mangrove
- Lagoon
- Quarry
- Sea
- Public Aquarium (i.e. work)
- Private Aquarium (i.e. home)
- Other: ______________________

44. When you felt this, what were you doing (wading, scuba, snorkel)?

*Mark only one oval.*

- Wading
- Scuba diving
- Snorkeling
- Working in an aquarium setting
- Other: ______________________

45. If you were close to a Cassiopea, how close to Cassiopea medusae were you?

*Mark only one oval.*

- <10 cm
- 10-50 cm
- 50-100 cm
- 1-2 m
- 2-5 m
- > 5 m
- Didn't see any or do not know
- Wasn't close to a Cassiopea

46. Same as above but for any kind of jellyfish. Provide species in next question if certain.

*Mark only one oval.*

- <10 cm
- 10-50 cm
- 50-100 cm
- 1-2 m
- 2-5 m
- >5 m

47. If non-Cassiopea jellyfish, provide genus or species name. Some common rhizostome jellyfish are provided in the images above.

48. To the best of your knowledge, how many Cassiopea (or other target Rhizostome jellyfish) were there in a 2 m radius of you?

*Mark only one oval.*

- 0
- 1
- 2-5
- 5-10
- 10-20
- 20+

49. Provide an average size estimate of medusae in your immediate vicinity. (2 m radius)

*Mark only one oval.*

- <5 cm
- 5-10 cm
- 10-15 cm
- 15-30 cm
- Other: ______________________

50. What was the highest Cassiopea (or other target Rhizostome jellyfish) density you saw in the area in which you were working (individuals/sq. meter)?

______________________

51. How far were you from this area of highest density?

*Mark only one oval.*

- <1 m
- 1-2 m
- 2-5 m
- 5-8 m
- >8 m
- Didn't see any or do not know
- No rhizostome jellyfish in the area

52. Did you engage in any action that resulted in disturbance or dislocation of medusae? This includes poking, kicking up surrounding water with fins, stepping on, etc.

*Mark only one oval.*

- Yes
- No
- Maybe

53. How long did you stay in this area?

*Mark only one oval.*

- <5 min
- 5-10 min
- 10-15 min
- 15 -30 min
- 30-60 min
- 1 hr+

54. What level of discomfort did you experience? (1- mild tickle, 3- burn and irritation, 5- severe pain)

*Mark only one oval.*

| | 1 | 2 | 3 | 4 | 5 | |
|---|---|---|---|---|---|---|
| Mild tickle | ◯ | ◯ | ◯ | ◯ | ◯ | Severe pain |

55. What would you consider this discomfort most comparable to?

_______________________________

56. Did you experience any skin discoloration in this area post-discomfort?

*Mark only one oval.*

- ◯ Yes
- ◯ No
- ◯ Don't know

57. Is there anything else you would like to tell us about this instance?

_______________________________
_______________________________
_______________________________
_______________________________
_______________________________

## Permissions for Use

Thank you for your responses. Before you submit, please select the option below for release of this information.

58. Permissions: Do you consent to the use of your survey answers in a public journal communication on experiences of contactless jellyfish stings? *

*Mark only one oval.*

- No.
- Yes, you may use my answers as data points.
- Yes, you may use my answers as data points and my anonymous written responses.
- Yes, you may use my answers as data points and my written responses, please provide my name separately in acknowledgments.

59. Name for Acknowledgement Section: Thank you for your responses.

This content is neither created nor endorsed by Google.

Google Forms
