# Supplementary material for: Raising Awareness of the Severity of “Contactless Stings” by Cassiopea Jellyfish and Kin
Source: Animals (Basel). 2021 Nov 24;11(12):3357. doi: 10.3390/ani11123357 (PMC8698115; doi:10.3390/ani11123357)
Supplement: Supplementary file 1 [file animals-11-03357-s001.zip › animals-1434736-supplementary/animals-1434736-Proofed Supplementary/Surveys/Stinging Water Survey (French).pdf]

Enquête sur l'eau urticante

L'"eau urticante" est la sensation d'inconfort inattendu que ressentent les plongeurset les pêcheurs dans les zones où se trouve la méduse Cassiopée. Ce phénomène est digne d'attention car les personnes interrogées rapportent souvent ne pas avoir été en contact direct avec les méduses. En général, l'irritation se limite à la peau exposée qui est submergée. Comme l'indique un article récent paru dans Nature Communications Biology (https://www.nature.com/articles/s42003-020-0777-8), les méduses Cassiopée produisent un mucus riche en structures contenant des nématocystes, connues sous le nom de "cassiosomes", qui provoquent une piqûre inconfortable voire douloureuse, appelée "eau urticante". Cette étude est menée par certains des auteurs de cet article (Kaden Muffett, Anna Klompen, Cheryl Ames et Allen Collins) afin de déterminer dans quelles situations les sondés expérimentent ce phénomène d'"eau urticante" et la gamme de réactions physiques qui en résultent. Cette enquête est menée avec l'intention explicite de la publier dans une revue scientifique. Le compte rendu d'une expérience individuelle devrait prendre environ 6 à 8 minutes. Si vous avez eu plus d'une expérience, nous vous encourageons à utiliser les trois sections de ce formulaire et à rapporter jusqu'à trois expériences. Bien que nous ne recueillions ni ne publiions d'informations personnelles, comme pour toute enquête en ligne, nous ne pouvons pas garantir la confidentialité intégrale de vos réponses, car l'hôte de l'enquête conserve certains privilèges d'accès.

* Required

Titre de l'étude de recherche : Enquête sur les expositions à l'eau urticante
Enquêteur : Maria Pia Miglietta

Pourquoi me demande-t-on de participer à cette étude ?
Vous êtes invité à participer à cette étude parce que nous essayons d'en savoir plussur les expériences des chercheurs et des aquariophiles concernant le phénomène d'eau urticante impliquant des méduses rhizostomes.

Vous avez été sélectionné comme participant potentiel à cette étude parce que vous avez répondu à notre demande de volontaires par e-mail. Vous devez être âgéde 18 ans ou plus pour participer à cette étude.

Pourquoi cette enquête est-elle menée ?
L'étude est conçue pour déterminer les situations qui conduisent à des expériences réelles d'"eau urticante" et la gamme de réactions physiques qui en résultent. Cetteenquête est créée dans l'intention de publier un bref communiqué dans le domaine de la biologie sur ce sujet.

Quelle est la durée de l'enquête ?
Elle prendra environ 7 à 30 minutes de votre temps en fonction du nombre d'expériences que vous souhaitez enregistrer.

Que se passe-t-il si je dis "Oui, je veux participer à cette enquête" ?
Si vous décidez de participer, veuillez sélectionner "J'accepte" à la fin de cette section.

Que se passe-t-il si je ne veux pas participer à cette recherche ?
Votre participation à cette étude est volontaire. Vous pouvez décider de ne pas participer à cette enquête et cela ne sera pas retenu contre vous. Vous pouvez quitter

Informed Consent

l'étude à tout moment.

Ma participation à cette étude pourrait-elle me nuire ?
Il n'y a pas de questions sensibles dans cette enquête qui pourraient causer un malaise. Toutefois, vous pouvez sauter toute question à laquelle vous ne souhaitez pas répondre ou quitter l'enquête à tout moment.

Qu'advient-il des informations recueillies dans le cadre de l'étude ?
Vous pouvez consulter la politique de confidentialité de l'hôte de l'enquête à l'adresse suivante : https://policies.google.com/privacyAucun identifiant personnel direct ne sera collecté à moins que vous ne demandiez volontairement à être inclus dans les remerciements.

Vos informations resteront confidentielles dans la mesure où la loi le permet. Les résultats de l'étude peuvent être publiés, mais votre identité restera confidentielle.

À qui puis-je m'adresser ?
N'hésitez pas à poser des questions concernant cette étude. Vous pouvez contacter Kade Muffett maintenant ou plus tard si vous avez d'autres questions ou préoccupations au 202-368-8338 et à kmmuffett@tamu.edu. Vous pouvez également contacter le Human Research Protection Program de la Texas A&M University (qui est un groupe de personnes examinant les recherches afin de protéger vos droits) par téléphone au 1-979-458-4067, par téléphone au 1-855-795-8636 ou par courriel à irb@tamu.edu pour :

- une aide supplémentaire pour toute question concernant cette enquête
- exprimer des préoccupations ou des plaintes concernant cette enquête
- obtenir des réponses aux questions sur vos droits en tant que participant à l'enquête
- des préoccupations au cas où le personnel de recherche ne pourrait être joint
- le désir de parler à quelqu'un d'autre que le personnel de recherche

Si vous voulez une copie de ce consentement pour vos dossiers, vous pouvez l'imprimer à partir de l'écran.
Si vous souhaitez participer, veuillez cliquer sur le bouton "J'accepte" et vous serez dirigé vers l'enquête.
Si vous ne souhaitez pas participer à cette étude, veuillez sélectionner "Je refuse" ou sélectionnez X dans le coin de votre navigateur

1. Acceptez-vous de participer à cette enquête ? *

Mark only one oval.

- [ ] J’accepte
- [ ] Je refuse

Informations générales

Veuillez fournir quelques informations afin que nous puissions mieux classer vos expériences.

2. Depuis combien de temps (au total) travaillez-vous avec des méduses Cassiopée ouautres rhizostomes ?

Mark only one oval.

- Je n'ai pas travaillé avec ces méduses à des fins de recherche ou d'aquariophilie
- < 1 an
- 1 à 3 ans
- 3 à 6 ans
- 6 ans et plus

3. Combien de fois avez-vous senti "l'eau urticante" ?

Mark only one oval.

- Une fois
- Deux fois
- Trois fois
- Plus de trois fois
- Jamais *Skip to question 59*

4. J'ai fait l'expérience de «l'eau urticante» en tant que :

Check all that apply.

- [ ] Aquariophile professionnel
- [ ] Propriétaire d'un aquarium domestique
- [ ] Chercheur (y compris les étudiants en cours de master ou de thèse)
- [ ] Étudiant (en cours de licence)
- [ ] Nageur récréatif/plongeur (masque et tuba), lors d'autres activités de loisirs
- [ ] Autre

Expérience 1

Veuillez ne détailler ici qu'une seule expérience

5. Où géographiquement (aussi précisément que possible) avez-vous ressenti "l'eau urticante" ?

6. Si vous connaissez les coordonnées de ce lieu, veuillez les inclure ci-dessous, sinon passez cette question.

7. Comment classifieriez-vous ce lieu

Mark only one oval.

- Mangrove
- Lagon
- Carrière
- Mer
- Aquarium public (c'est-à-dire travail)
- Aquarium privé (c'est-à-dire à domicile)
- Autre

8. Quand vous avez ressenti cela, que faisiez-vous (baignade, plongée, tuba) ?

Mark only one oval.

- Baignade en eau peu profonde
- Plongée sous-marine
- Plongée avec masque et tuba
- Travailler dans un aquarium
- Autre

9. Si vous étiez proche d'une Cassiopée, à quel point étiez-vous proche de la méduse Cassiopée ?

Mark only one oval.

- <10 cm
- 10-50 cm
- 50-100 cm
- 1-2 m
- 2-5 m
- > 5 m
- Option 7
- Je n'en ai pas vu ou je ne sais pas
- N'était pas proche d'une Cassiopé

10. Pareil que ci-dessus, mais pour n'importe quel type de méduse. Indiquez les espèces dans la question suivante si vous en êtes certain

Mark only one oval.

- <10 cm
- 10-50 cm
- 50-100 cm
- 1-2 m
- 2-5 m
- >5 m

11. S'il s'agit d'une méduse autre que Cassiopée, indiquez le nom du genre ou de l'espèce. Certaines méduses rhizostomes communes sont présentées dans les images ci-dessus.

____

12. À votre connaissance, combien de méduses Cassiopée (ou autres méduses rhizostomes) étaient présentes dans un rayon de 2 m autour de vous ?

Mark only one oval.

- 0
- 1
- 2-5
- 5-10
- 10-20
- 20+

13. Fournissez une estimation de la taille moyenne des méduses à proximité immédiate. (Dans un rayon de 2 m)

Mark only one oval.

- <5 cm
- 5-10 cm
- 10-15 cm
- 15-30 cm
- Other: ______________________

14. Quelle était la plus forte densité de Cassiopées (ou autres méduses rhizostomes) que vous avez vue dans la zone dans laquelle vous vous trouviez (individus/mètre carré) ?

____

15. A quelle distance étiez-vous de cette zone de plus forte densité ?

Mark only one oval.

- <1 m
- 1-2 m
- 2-5 m
- 5-8 m
- >8 m
- Je n'en ai pas vu ou je ne sais pas
- Aucune méduse à rhizostome dans la région

16. Avez-vous entrepris une action qui a entraîné une perturbation ou un éparpillement des méduses ? Il s'agit notamment de pousser, de donner des coups de pied dans l'eau environnante avec des palmes, de marcher dans l'eau, etc.

Mark only one oval.

- Oui
- Non
- Peut-être

17. Combien de temps êtes-vous resté dans cet endroit ?

Mark only one oval.

- <5 min
- 5-10 min
- 10-15 min
- 15 -30 min
- 30-60 min
- 1 heure et plus

18. Quel niveau d'inconfort avez-vous ressenti ? (1- picotement léger, 3- brûlure et irritation, 5- douleur intense)

Mark only one oval.

	1	2	3	4	5	
Picotement léger						Douleur intense

19. À quoi considérez-vous que ce malaise soit le plus comparable ?

20. Avez-vous constaté une décoloration de la peau dans cette zone après l'incident ?

Mark only one oval.

- Oui
- Non
- Je ne sais pas

21. Y a-t-il autre chose que vous voudriez rajouter à propos de cet incident ?

22. Avez-vous une autre expérience que vous souhaitez détailler ?

Mark only one oval.

- Oui (En cliquant sur oui, vous pourrez répéter ces questions pour une expérience supplémentaire)
- Non (En cliquant sur non, vous serez dirigé vers les autorisations d'utilisation et la soumission finale) *Skip to question 59*

Expérience 2

Veuillez ne détailler ici qu'une seule expérience

23. Où géographiquement (aussi précisément que possible) avez-vous ressenti "l'eau urticante" ?

24. Si vous connaissez les coordonnées de ce lieu, veuillez les inclure ci-dessous, sinon passez cette question.

25. Comment classifieriez-vous ce lieu

Mark only one oval.

- Mangrove
- Lagon
- Carrière
- Mer
- Aquarium public (c'est-à-dire travail)
- Aquarium privé (c'est-à-dire à domicile)
- Autre

26. Quand vous avez ressenti cela, que faisiez-vous (baignade, plongée, tuba) ?

Mark only one oval.

- Baignade en eau peu profonde
- Plongée sous-marine
- Plongée avec masque et tuba
- Travailler dans un aquarium
- Autre

27. Si vous étiez proche d'une Cassiopée, à quel point étiez-vous proche de la méduse Cassiopée ?

Mark only one oval.

- <10 cm
- 10-50 cm
- 50-100 cm
- 1-2 m
- 2-5 m
- > 5 m
- Option 7
- Je n'en ai pas vu ou je ne sais pas
- N'était pas proche d'une Cassiopé

28. Pareil que ci-dessus, mais pour n'importe quel type de méduse. Indiquez les espèces dans la question suivante si vous en êtes certain

Mark only one oval.

- <10 cm
- 10-50 cm
- 50-100 cm
- 1-2 m
- 2-5 m
- >5 m

29. S'il s'agit d'une méduse autre que Cassiopée, indiquez le nom du genre ou de l'espèce. Certaines méduses rhizostomes communes sont présentées dans les images ci-dessus.

____

30. À votre connaissance, combien de méduses Cassiopée (ou autres méduses rhizostomes) étaient présentes dans un rayon de 2 m autour de vous ?

Mark only one oval.

- 0
- 1
- 2-5
- 5-10
- 10-20
- 20+

31. Fournissez une estimation de la taille moyenne des méduses à proximité immédiate. (Dans un rayon de 2 m)

Mark only one oval.

- <5 cm
- 5-10 cm
- 10-15 cm
- 15-30 cm
- Other: ______________________

32. Quelle était la plus forte densité de Cassiopées (ou autres méduses rhizostomes) que vous avez vue dans la zone dans laquelle vous vous trouviez (individus/mètre carré) ?

____

33. A quelle distance étiez-vous de cette zone de plus forte densité ?

Mark only one oval.

- <1 m
- 1-2 m
- 2-5 m
- 5-8 m
- >8 m
- Je n'en ai pas vu ou je ne sais pas
- Aucune méduse à rhizostome dans la région

34. Avez-vous entrepris une action qui a entraîné une perturbation ou un éparpillement des méduses ? Il s'agit notamment de pousser, de donner des coups de pied dans l'eau environnante avec des palmes, de marcher dans l'eau, etc.

Mark only one oval.

- Oui
- Non
- Peut-être

35. Combien de temps êtes-vous resté dans cet endroit ?

Mark only one oval.

- <5 min
- 5-10 min
- 10-15 min
- 15 -30 min
- 30-60 min
- 1 heure et plus

36. Quel niveau d'inconfort avez-vous ressenti ? (1- picotement léger, 3- brûlure et irritation, 5- douleur intense)

Mark only one oval.

	1	2	3	4	5	
Picotement léger						Douleur intense

37. À quoi considérez-vous que ce malaise soit le plus comparable ?

38. Avez-vous constaté une décoloration de la peau dans cette zone après l'incident ?

Mark only one oval.

- Oui
- Non
- Je ne sais pas

39. Y a-t-il autre chose que vous voudriez rajouter à propos de cet incident ?

40. Avez-vous une autre expérience que vous souhaitez détailler ?

Mark only one oval.

- Oui (En cliquant sur oui, vous pourrez répéter ces questions pour une expérience supplémentaire)
- Non (En cliquant sur non, vous serez dirigé vers les autorisations d'utilisation et la soumission finale) *Skip to question 59*

Expérience 3

Veuillez ne détailler ici qu'une seule expérience

41. Où géographiquement (aussi précisément que possible) avez-vous ressenti "l'eau urticante" ?

42. Si vous connaissez les coordonnées de ce lieu, veuillez les inclure ci-dessous, sinon passez cette question.

43. Comment classifieriez-vous ce lieu

Mark only one oval.

- Mangrove
- Lagon
- Carrière
- Mer
- Aquarium public (c'est-à-dire travail)
- Aquarium privé (c'est-à-dire à domicile)
- Autre

44. Quand vous avez ressenti cela, que faisiez-vous (baignade, plongée, tuba) ?

Mark only one oval.

- Baignade en eau peu profonde
- Plongée sous-marine
- Plongée avec masque et tuba
- Travailler dans un aquarium
- Autre

45. Si vous étiez proche d'une Cassiopée, à quel point étiez-vous proche de la méduse Cassiopée ?

Mark only one oval.

- <10 cm
- 10-50 cm
- 50-100 cm
- 1-2 m
- 2-5 m
- > 5 m
- Option 7
- Je n'en ai pas vu ou je ne sais pas
- N'était pas proche d'une Cassiopé

46. Pareil que ci-dessus, mais pour n'importe quel type de méduse. Indiquez les espèces dans la question suivante si vous en êtes certain

Mark only one oval.

- <10 cm
- 10-50 cm
- 50-100 cm
- 1-2 m
- 2-5 m
- >5 m

47. S'il s'agit d'une méduse autre que Cassiopée, indiquez le nom du genre ou de l'espèce. Certaines méduses rhizostomes communes sont présentées dans les images ci-dessus.

____

48. À votre connaissance, combien de méduses Cassiopée (ou autres méduses rhizostomes) étaient présentes dans un rayon de 2 m autour de vous ?

Mark only one oval.

- 0
- 1
- 2-5
- 5-10
- 10-20
- 20+

49. Fournissez une estimation de la taille moyenne des méduses à proximité immédiate. (Dans un rayon de 2 m)

Mark only one oval.

- <5 cm
- 5-10 cm
- 10-15 cm
- 15-30 cm
- Other: ______________________

50. Quelle était la plus forte densité de Cassiopées (ou autres méduses rhizostomes) que vous avez vue dans la zone dans laquelle vous vous trouviez (individus/mètre carré) ?

____

51. A quelle distance étiez-vous de cette zone de plus forte densité ?

Mark only one oval.

- <1 m
- 1-2 m
- 2-5 m
- 5-8 m
- >8 m
- Je n'en ai pas vu ou je ne sais pas
- Aucune méduse à rhizostome dans la région

52. Avez-vous entrepris une action qui a entraîné une perturbation ou un éparpillement des méduses ? Il s'agit notamment de pousser, de donner des coups de pied dans l'eau environnante avec des palmes, de marcher dans l'eau, etc.

Mark only one oval.

- Oui
- Non
- Peut-être

53. Combien de temps êtes-vous resté dans cet endroit ?

Mark only one oval.

- <5 min
- 5-10 min
- 10-15 min
- 15 -30 min
- 30-60 min
- 1 heure et plus

54. Quel niveau d'inconfort avez-vous ressenti ? (1- picotement léger, 3- brûlure et irritation, 5- douleur intense)

Mark only one oval.

	1	2	3	4	5	
Picotement léger						Douleur intense

55. À quoi considérez-vous que ce malaise soit le plus comparable ?

56. Avez-vous constaté une décoloration de la peau dans cette zone après l'incident ?

Mark only one oval.

- Oui
- Non
- Je ne sais pas

57. Y a-t-il autre chose que vous voudriez rajouter à propos de cet incident ?

58. Avez-vous une autre expérience que vous souhaitez détailler ?

Mark only one oval.

- Oui (En cliquant sur oui, vous pourrez répéter ces questions pour une expérience supplémentaire)
- Non (En cliquant sur non, vous serez dirigé vers les autorisations d'utilisation et la soumission finale) *Skip to question 59*

Autorisations d'utilisation

Merci pour vos réponses. Avant de soumettre votre formulaire, veuillez sélectionner l'option ci-dessous pour la diffusion de ces informations.

59. Autorisations : Consentez-vous à l'utilisation de vos réponses à l'enquête dans un journal public sur les expériences de piqûres de méduses sans contact ? *

Mark only one oval.

- ( ) Non.
- ( ) Oui, vous pouvez utiliser mes réponses comme points de données.
- ( ) Oui, vous pouvez utiliser mes réponses comme points de données et mes réponses écrites de manière anonyme.
- ( ) Oui, vous pouvez utiliser mes réponses comme points de données et mes réponses écrites, veuillez indiquer mon nom séparément dans les remerciements.

60. Nom de la section des remerciements: Merci pour vos réponses.

____

This content is neither created nor endorsed by Google.

Google Forms
