# Supplementary material for: Raising Awareness of the Severity of “Contactless Stings” by Cassiopea Jellyfish and Kin
Source: Animals (Basel). 2021 Nov 24;11(12):3357. doi: 10.3390/ani11123357 (PMC8698115; doi:10.3390/ani11123357)
Supplement: Supplementary file 1 [file animals-11-03357-s001.zip › animals-1434736-supplementary/animals-1434736-Proofed Supplementary/Surveys/Stinging Water Survey (Japanese).pdf]

刺傷水の調査

「刺傷水」は、サカサクラゲ属のクラゲが生息する地域でシュノーケリングやウェーディングをする人が経験する予期せぬ不快感です。クラゲにまったく触れていないという報告が多いので、これは注目に値します。一般的に、刺激は水中で露出した皮膚に限定されます。Nature Communications Biologyの最近の論文（https://www.nature.com/articles/s42003-020-0777-8）で報告されているように、サカサクラゲ属のクラゲは「カシオソーム」として知られる刺胞を含む構造を大量に含む粘液を生成します。その結果、「刺傷水」として知られる不快な痛みを伴う刺し傷が生じます。この調査は、この論文の著者の何人か（Kaden Muffett、Anna Klompen、Cheryl Ames、Allen Collins）によって実施され、「刺傷水」の実際の体験につながる状況と、結果として生じるさまざまな物理的反応を特定します。本調査は、科学雑誌に掲載することを明確に意図して配布されています。個々の経験の報告には、約6〜8分かかります。複数の経験がある場合は、このフォーム内の3つの入力セクションすべてを使用して、最大3つの経験を報告することをお勧めします。他のオンライン調査と同様に、個人情報を収集または公開していませんが、調査のホストは一部のアクセス権限を保持しているため、回答の完全なプライバシーを保証することはできません。

* Required

インフォームド・コンセント

調査研究のタイトル：刺傷水相互作用調査
調査員：Maria Pia Miglietta

なぜ、この調査研究への参加を求められているのですか？
根口クラゲの周りの刺傷水現象に関する研究者や水族館の経験についてもっと学びたいと思っているので、本研究に参加するよう招待されています。

あなたはボランティアの電子メール要求に応答したため、この調査の参加候補者として選ばれました。参加するには18歳以上である必要があります。

なぜ、この研究が行われているのですか？
この調査は、「刺傷水」の実際の体験につながる状況と、その結果生じるさまざまな物理的反応を特定することを目的としています。この調査は、このトピックに関するコミュニケーション生物学の短い招待されたコミュニケーションを公開するという明確な意図を持って作成されています。

時間はどのくらいかかりますか？
記録したい体験の数にもよりますが、7分から30分ほどかかります。

「はい、この研究に参加したい」と言ったらどうなりますか？
参加する場合は、このセクションの最後にある「同意する」を選択してください。

本研究に参加したくない場合はどうなりますか？
本研究への参加は任意です。研究へ参加するかどうかを決めることができ、参加者に対して拘束することはありません。いつでも調査を離れることができます。

本研究に参加することで参加者に害を及ぼす可能性はありますか？
本調査には、不快感を引き起こす可能性のあるデリケートな質問はありません。ただし、回答したくない質問はスキップするか、いつでもアンケートを終了することができます。

調査のために収集された情報はどうなりますか？
調査ホストの機密保持ポリシーは、https：//policies.google.com/privacyで確認できます。
謝辞に含めるように自発的に要求しない限り、直接の個人IDは収集されません。

参加者の情報は、法律で許可されている範囲で秘密にされます。調査研究の結果は公開される場合がありますが、参加者の身元は秘密にされます。

誰と話せますか?
本研究についてお気軽にご質問ください。 202-368-8338およびkmmuffett@tamu.eduで追加の質問または懸念がある場合は、現在または後でKade Muffettに連絡することができます。
また、テキサスA&M大学（あなたの権利を保護するために研究をレビューする人々のグループ）のヒューマンリサーチプロテクションプログラムに電話1-979-458-4067、フリーダイヤル1-855-795-8636で連絡することもできます。、またはirb@tamu.eduに電子メールで:
•研究に関する質問についての追加のヘルプ
•研究に関する懸念や苦情を表明する
•研究参加者としての参加者の権利に関する質問への回答を得る
•研究スタッフに連絡が取れなかった場合の懸念
•研究スタッフ以外の人と話したいという願望

記録のためにこの同意書のコピーが必要な場合は、画面から印刷できます。
参加をご希望の場合は、「同意する」ボタンをクリックしてください。アンケートにご案内します。

この調査に参加したくない場合は、「同意しない」を選択するか、ブラウザの隅にあるXを選択してください。

1. この研究に参加することに同意しますか? *

Mark only one oval.

- 同意します
- 同意しません

一般的な情報

私たちがあなたの経験をよりよく分類できるように、いくつかの情報を提供してください。

2. サカサクラゲ属または他の根口クラゲ類とどのくらい（合計）接してきましたか?

Mark only one oval.

- 私はこれらのクラゲを研究や水族館の目的で扱ったことがありません
- 1年未満
- 1～3年
- 3～6年
- 6年より長い

3. 「刺傷水」を何回感じましたか?

Mark only one oval.

- 1回
- 2回
- 3回
- 3回より多い
- 感じたことはない　*Skip to question 58*

4. 私は以下の仕事または生活をしている間、刺傷水を経験しました:

Check all that apply.

- [ ] プロのアクアリスト
- [ ] ホームアクアリウムの所有者
- [ ] 研究者（大学院生を含む）
- [ ] 学生（大学院生を含まない）
- [ ] レジャー活動するレクリエーションスイマー/シュノーケラー

Other: [ ] ____________

経験 1

ここでは、1つの体験のみを詳しく説明してください。

5. 地理的に（可能な限り具体的に）どこで「刺傷水」を体感しましたか？

6. 刺傷水を体験した場所の座標がわかる場合は以下に記入して下さい。わからない場合はこの質問をスキップしてください。

7. 刺傷水を経験した場所はどこですか？

Mark only one oval.

- マングローブ
- 沼や池、礁湖
- 石切り場、採石場、源泉
- 海
- 公共の水族館（仕事場）
- プライベートアクアリウム（家）
- Other:

8. 刺傷水を経験したとき、何をしていましたか（水遊び、スキューバ、シュノーケル）？

Mark only one oval.

- ウェーディング
- スキューバダイビング
- シュノーケリング
- 水族館での作業
- Other: ____________________

9. サカサクラゲ属が近くにいた場合、あなたはクラゲとどれくらいの距離にいましたか?

Mark only one oval.

- 10 cmより近い
- 10 ~ 50 cm
- 50 ~ 100 cm
- 1 ~ 2 m
- 2 ~ 5 m
- 5 mより遠い
- サカサクラゲ属は見えなかった、あるいはいたかどうかわからなかった
- サカサクラゲ属の近くにいなかった

10. 上記と同じですが、あらゆる種類のクラゲについての質問です。記憶が確かであれば、次の質問でクラゲの種類をお答えください。

Mark only one oval.

- 10 cmより近い
- 10 ~ 50 cm
- 50 ~ 100 cm
- 1 ~ 2 m
- 2 ~ 5 m
- 5 mより遠い

11. サカサクラゲ以外のクラゲの場合は、属または種の名前を入力してください。 いくつかの一般的な根口クラゲは上の画像でご確認ください。

____

12. あなたの知る限り、あなたの半径2 mの中に、サカサクラゲ属（または他の根口クラゲ目）が何匹いましたか?

Mark only one oval.

- 0匹
- 1匹
- 2 ~ 5匹
- 5 ~ 10匹
- 10 ~ 20匹
- 20匹以上

13. すぐ近く（半径2m）にいたクラゲの平均サイズはどのくらいですか?

Mark only one oval.

- 5 cmより小さい
- 5 ~ 10 cm
- 10 ~ 15 cm
- 15 ~ 30 cm
- Other: ______________________

14. あなたが生活や仕事をしていた地域で見たサカサクラゲ属（または他の根口クラゲ目）生息密度（個体数/平方メートル）は最大でどのくらいでしたか?

____

15. あなたはそのとき、最大の個体数密度の場所からどれくらい離れていましたか?

Mark only one oval.

- 1 mより近い
- 1 ~ 2 m
- 2 ~ 5 m
- 5 ~ 8 m
- 8 mより遠い
- クラゲは見えなかった、あるいはいたかどうかわからなかった
- この地域には根口クラゲ目はいない

16. クラゲをかき混ぜたり、動かしたりするような行動をしましたか？ これには、突く、フィンで周囲の水を蹴る、踏むなどが含まれます。

Mark only one oval.

- はい
- いいえ
- おそらくクラゲを動かした

17. この地域にどのくらい滞在しましたか？

Mark only one oval.

- 5分より短い
- 5～10分
- 10～15分
- 15～30分
- 30～60分
- 1時間以上

18. どの程度の不快感を経験しましたか？ (1-軽くチクチクする程度、3-火傷と刺激、5-激しい痛み)

Mark only one oval.

	1	2	3	4	5	
軽くチクチクする程度						激しい痛み

19. この不快感は何に例えられますか？

20. 不快感の後、皮膚の変色を経験しましたか?

Mark only one oval.

- はい
- いいえ
- わからない

21. この事例について他に何か伝えたいことはありますか?

22. あなたが詳しく伝えたい別の経験がありますか?

Mark only one oval.

- はい ([はい]をクリックすると、これらの質問を繰り返して追加のエクスペリエンスを得ることができます)
- いいえ ([いいえ]をクリックすると、使用許可と最終提出に移動します) *Skip to question 58*

経験 2

ここでは、1つの体験のみを詳しく説明してください。

23. 地理的に(可能な限り具体的に)どこで「刺傷水」を経験しましたか?

24. 刺傷水を体験した場所の座標がわかる場合は以下に記入して下さい。わからない場合はこの質問をスキップしてください。

____

25. 刺傷水を経験した場所はどこですか?

Mark only one oval.

- ◯ マングローブ
- ◯ 沼や池、礁湖
- ◯ 石切り場、採石場、源泉
- ◯ 海
- ◯ 公共の水族館（仕事場）
- ◯ プライベートアクアリウム（家）
- ◯ Other: _______________________________

26. 刺傷水を経験したとき、何をしていましたか（水遊び、スキューバ、シュノーケル）？

Mark only one oval.

- ◯ ウェーディング
- ◯ スキューバダイビング
- ◯ シュノーケリング
- ◯ 水族館での作業
- ◯ Other: _______________________________

27. サカサクラゲ属が近くにいた場合、あなたはクラゲとどれくらいの距離にいましたか?

Mark only one oval.

- 10 cmより近い
- 10 ~ 50 cm
- 50 ~ 100 cm
- 1 ~ 2 m
- 2 ~ 5 m
- 5 mより遠い
- サカサクラゲ属は見えなかった、あるいはいたかどうかわからなかった
- サカサクラゲ属の近くにいなかった

28. 上記と同じですが、あらゆる種類のクラゲについての質問です。記憶が確かであれば、次の質問でクラゲの種類をお答えください。

Mark only one oval.

- 10 cmより近い
- 10 ~ 50 cm
- 50 ~ 100 cm
- 1 ~ 2 m
- 2 ~ 5 m
- 5 mより遠い

29. サカサクラゲ以外のクラゲの場合は、属または種の名前を入力してください。 いくつかの一般的な根口クラゲは上の画像でご確認ください。

____

30. あなたの知る限り、あなたの半径2 mの中に、サカサクラゲ属（または他の根口クラゲ目）が何匹いましたか?

Mark only one oval.

- 0匹
- 1匹
- 2～5匹
- 5～10匹
- 10～20匹
- 20匹以上

31. すぐ近く（半径2m）にいたクラゲの平均サイズはどのくらいですか?

Mark only one oval.

- 5 cmより小さい
- 5～10 cm
- 10～15 cm
- 15～30 cm
- Other: ____________________

32. あなたが生活や仕事をしていた地域で見たサカサクラゲ属（または他の根口クラゲ目）生息密度（個体数/平方メートル）は最大でどのくらいでしたか?

____

33. あなたはそのとき、最大の個体数密度の場所からどれくらい離れていましたか?

Mark only one oval.

- 1 mより近い
- 1～2 m
- 2～5 m
- 5～8 m
- 8 mより遠い
- クラゲは見えなかった、あるいはいたかどうかわからなかった
- この地域には根口クラゲ目はいない

34. クラゲをかき混ぜたり、動かしたりするような行動をしましたか? これには、突く、フィンで周囲の水を蹴る、踏むなどが含まれます。

Mark only one oval.

- はい
- いいえ
- おそらくクラゲを動かした

35. この地域にどのくらい滞在しましたか?

Mark only one oval.

- 5分より短い
- 5～10分
- 10～15分
- 15～30分
- 30～60分
- 1時間以上

36. どの程度の不快感を経験しましたか？ (1-軽くチクチクする程度、3-火傷と刺激、5-激しい痛み)

Mark only one oval.

	1	2	3	4	5	
軽くチクチクする程度	◯	◯	◯	◯	◯	激しい痛み

37. この不快感は何に例えられますか?

____

38. 不快感の後、皮膚の変色を経験しましたか?

Mark only one oval.

◯ はい
◯ いいえ
◯ わからない

39. この事例について他に何か伝えたいことはありますか?

____
____
____
____
____

40. あなたが詳しく伝えたい別の経験がありますか?

Mark only one oval.

- はい([はい]をクリックすると、これらの質問を繰り返して追加のエクスペリエンスを得ることができます)
- いいえ([いいえ]をクリックすると、使用許可と最終提出に移動します) *Skip to question 58*

経験 3

ここでは、1つの体験のみを詳しく説明してください。

41. 地理的に(可能な限り具体的に)どこで「刺傷水」を経験しましたか?

42. 刺傷水を体験した場所の座標がわかる場合は以下に記入して下さい。わからない場合はこの質問をスキップしてください。

43. 刺傷水を経験した場所はどこですか?

Mark only one oval.

- マングローブ
- 沼や池、礁湖
- 石切り場、採石場、源泉
- 海
- 公共の水族館（仕事場）
- プライベートアクアリウム（家）
- Other: ______________

44. 刺傷水を経験したとき、何をしていましたか（水遊び、スキューバ、シュノーケル）？

Mark only one oval.

- ウェーディング
- スキューバダイビング
- シュノーケリング
- 水族館での作業
- Other: ______________

45. サカサクラゲ属が近くにいた場合、あなたはクラゲとどれくらいの距離にいましたか?

Mark only one oval.

- 10 cmより近い
- 10 ~ 50 cm
- 50 ~ 100 cm
- 1 ~ 2 m
- 2 ~ 5 m
- 5 mより遠い
- サカサクラゲ属は見えなかった、あるいはいたかどうかわからなかった
- サカサクラゲ属の近くにいなかった

46. 上記と同じですが、あらゆる種類のクラゲについての質問です。記憶が確かであれば、次の質問でクラゲの種類をお答えください。

Mark only one oval.

- 10 cmより近い
- 10 ~ 50 cm
- 50 ~ 100 cm
- 1 ~ 2 m
- 2 ~ 5 m
- 5 mより遠い

47. If non-Cassiopea jellyfish, provide genus or species name. Some common rhizostome jellyfish are provided in the images above.

48. サカサクラゲ以外のクラゲの場合は、属または種の名前を入力してください。 いくつかの一般的な根口クラゲは上の画像でご確認ください。

Mark only one oval.

- 0匹
- 1匹
- 2～5匹
- 5～10匹
- 10～20匹
- 20匹以上

49. すぐ近く（半径2m）にいたクラゲの平均サイズはどのくらいですか?

Mark only one oval.

- 5 cmより小さい
- 5～10 cm
- 10～15 cm
- 15～30 cm
- Other: ____________

50. あなたが生活や仕事をしていた地域で見たサカサクラゲ属（または他の根口クラゲ目）生息密度（個体数/平方メートル）は最大でどのくらいでしたか?

____

51. あなたはそのとき、最大の個体数密度の場所からどれくらい離れていましたか?

Mark only one oval.

- 1 mより近い
- 1～2 m
- 2～5 m
- 5～8 m
- 8 mより遠い
- クラゲは見えなかった、あるいはいたかどうかわからなかった
- この地域には根口クラゲ目はいない

52. クラゲをかき混ぜたり、動かしたりするような行動をしましたか? これには、突く、フィンで周囲の水を蹴る、踏むなどが含まれます。

Mark only one oval.

- はい
- いいえ
- おそらくクラゲを動かした

53. この地域にどのくらい滞在しましたか?

Mark only one oval.

- 5分より短い
- 5～10分
- 10～15分
- 15～30分
- 30～60分
- 1時間以上

54. どの程度の不快感を経験しましたか？　(1-軽くチクチクする程度、3-火傷と刺激、5-激しい痛み)

Mark only one oval.

	1	2	3	4	5	
軽くチクチクする程度	⬭	⬭	⬭	⬭	⬭	激しい痛み

55. この不快感は何に例えられますか?

____

56. 不快感の後、皮膚の変色を経験しましたか?

Mark only one oval.

- ⬭ はい
- ⬭ いいえ
- ⬭ わからない

57. この事例について他に何か伝えたいことはありますか?

____

____

____

____

____

使用許可

ご回答ありがとうございます。 送信する前に、この情報を公開するために以下のオプションを選択してください。

58. 許可：一般雑誌の報道で非接触型のクラゲの刺傷経験に関する調査回答を使用することに同意しますか？ *

Mark only one oval.

- いいえ
- はい、私の回答をデータとして使用できます。
- はい、私の回答をデータおよび匿名の書面による回答として使用できます。
- はい、私の回答をデータおよび書面による回答として使用できます。謝辞に私の名前を個別に入力してください。

59. 謝辞に記入するお名前（希望される場合のみ）：ご回答ありがとうございます。

This content is neither created nor endorsed by Google.

Google Forms
