# Supplementary material for: Raising Awareness of the Severity of “Contactless Stings” by Cassiopea Jellyfish and Kin
Source: Animals (Basel). 2021 Nov 24;11(12):3357. doi: 10.3390/ani11123357 (PMC8698115; doi:10.3390/ani11123357)
Supplement: Supplementary file 1 [file animals-11-03357-s001.zip › animals-1434736-supplementary/animals-1434736-Proofed Supplementary/Surveys/Stinging Water Survey (Spanish).pdf]

Encuesta sobre "Agua urticante"

"Agua urticante" ("stinging water" en inglés) es la sensación de una molestia inesperada que experimentan los buceadores y los zancudos en áreas donde hay medusas de Cassiopea. Esto es conocido ya que las personas informan con frecuencia de no tocar en absoluto las medusas. Generalmente, solo se irrita la piel expuesta que está sumergida. Como se informó en un artículo reciente en Nature Communications Biology (https://www.nature.com/articles/s42003-020-0777-8), la medusa de Cassiopea produce un moco con una alta cantidad de estructuras que contienen nematocistos conocidas como "cassiosomas" que resulta en una incómoda y dolorosa picadura conocida como "agua urticante". Esta encuesta está siendo realizada por algunos de los autores de este artículo (Kaden Muffett, Anna Klompen, Cheryl Ames y Allen Collins) para determinar qué experiencias a día de hoy conducen la picadura de este "agua urticante" y el rango de reacciones físicas resultantes. Esta encuesta se distribuye con la intención de su publicación en una revista científica. El tiempo que se tarda en rellenar una única experiencia debería llevar aproximadamente de 6 a 8 minutos. Si tiene más de una, le recomendamos que utilice las tres secciones de entrada de este formulario y que informe hasta un total de tres experiencias. No recopilamos ni publicamos información personal, como en cualquier encuesta online, no podemos garantizar la privacidad total de sus respuestas, ya que el anfitrión de la encuesta conserva algunos privilegios de acceso.

* Required

Título del estudio de investigación: Encuesta de interacciones de agua urticante
Investigadora: Maria Pia Miglietta

¿Por qué se me pide que participe en este estudio de investigación?
Está invitado a participar en este estudio porque estamos tratando de aprender más sobre las experiencias de los investigadores y acuaristas con el fenómeno del agua urticante de las medusas rhizostomales.

Fue seleccionado como posible participante en este estudio porque respondió a nuestra solicitud de voluntarios por correo electrónico. Debes tener 18 años o más para participar.

¿Por qué se realiza este estudio?
La encuesta está diseñada para determinar qué situaciones conducen a experiencias con "agua urticante" y la variedad de reacciones físicas resultantes. Esta encuesta se está realizando con la intención de publicar una breve comunicación invitada por la revista Biología de las Comunicaciones.

¿Cuánto tiempo dura la encuesta?
Le llevará entre 7 y 30 minutos de su tiempo, dependiendo de cuántas experiencias quiera registrar.

¿Qué sucede si digo "Sí, quiero participar en este estudio"?
Si decide participar, seleccione "Acepto" al final de esta sección

¿Qué pasa si no quiero participar en este estudio?
Tu participación en esta investigación es voluntaria. Puede decidir no

Consentimiento informado

participar y no se tomará en su contra. Puede abandonar la encuesta en cualquier momento.

¿Hay alguna forma que pueda perjudicarme al participar en este estudio ?
En esta encuesta no hay preguntas delicadas que deberían sentirse incómodo. Sin embargo, puede omitir cualquier pregunta que no desee responder o salir de la encuesta en cualquier momento.

¿Qué sucede con la información recopilada para este estudio?
Puede ver la política de confidencialidad del anfitrión de la encuesta en: https://policies.google.com/privacy
No se recopilarán identificadores personales directos A MENOS que usted solicite voluntariamente ser incluido en los agradecimientos.

Sus datos se mantendrán confidenciales en la medida permitida por la ley. Los resultados del estudio pueden publicarse, pero su identidad se mantendrá confidencial.

¿Con quién puedo entrar en contacto?
No dude en hacer preguntas sobre este estudio. Puede comunicarse con Kade Muffett siempre que lo necesite si tiene alguna pregunta adicional al 202-368-8338 y kmmuffett@tamu.edu.
También puede entrar em contacto con el Programa de Protección de Investigaciones Humanas en Texas A&M University (que es un grupo de personas que revisan el estudio para proteger sus derechos) por teléfono al 1-979-458-4067, sin cargo al 1-855-795-8636 , o bien por correo electrónico a irb@tamu.edu para:

- ayuda adicional con cualquier pregunta sobre el estudio
- preocupaciones o quejas sobre la investigación
- obtener respuestas a preguntas sobre sus derechos como participante de una investigación
- en caso de no poder contactar con el personal de investigación
- hablar con otra persona que no sea el personal de investigación

Si desea una copia de este consentimiento, puede imprimirlo desde la pantalla.
Si desea participar, haga clic en el botón "Acepto".

Si no desea participar en este estudio, seleccione "No estoy de acuerdo" o seleccione X en la esquina de su navegador.

1. ¿Está de acuerdo en participar en este estudio? *

Mark only one oval.

- ( ) Acepto
- ( ) No estoy de acuerdo

Información general

Por favor proporcione alguna información para que podamos categorizar mejor sus experiencias.

2. ¿Por cuánto tiempo ha trabajado con Cassiopea u otra medusa Rhizostoma?

Mark only one oval.

- No he trabajado con estas medusas con fines de investigación o de acuario
- < 1 año
- 1-3 años
- 3-6 años
- 6+ años

3. ¿Cuántas veces ha sentido el "agua urticante"?

Mark only one oval.

- Una vez
- Dos veces
- Tres veces
- Más de tres
- Nunca *Skip to question 58*

4. Sentí el agua urticante mientras estaba como:

Check all that apply.

- [ ] Acuarista profesional
- [ ] En mi acuario personal
- [ ] Investigador/a (incluidos estudiantes de posgrado)
- [ ] Alumno/a (no graduado)
- [ ] Nadando/buceando, durante otras actividades de ócio

Other: [ ] ____________________

Experiencia 1

Por favor, describa aqui una experiencia.

5. ¿Dónde geográficamente (lo más específico posible) sintió “agua urticante”?

____
____
____
____
____

6. Si conoce las coordenadas de esta ubicación, inclúyalas a continuación; de lo contrario, omita esta pregunta.

____

7. Este lugar lo clasificaría como:

Mark only one oval.

- ◯ Manglar
- ◯ Laguna
- ◯ Presa
- ◯ Mar
- ◯ Acuario publico (i.e. trabajo)
- ◯ Acuario privado (i.e. casa)
- ◯ Other: _______________________

8. Cuando sintió esto, ¿qué estaba haciendo (vadear, bucear, snorkel)?

Mark only one oval.

- Caminar por el agua
- Bucear
- Snorkel
- Trabajar en un acuario
- Other: ____________________

9. Si estaba cerca de una Cassiopea, ¿qué tan cerca estaba de la medusa?

Mark only one oval.

- <10 cm
- 10-50 cm
- 50-100 cm
- 1-2 m
- 2-5 m
- > 5 m
- No vi ninguna o no lo sé
- No estaba cerca de una Cassiopea

10. Igual que la pregunta anterior, pero para cualquier tipo de medusa. Proporcione las especies en la siguiente pregunta si es necesario.

Mark only one oval.

- <10 cm
- 10-50 cm
- 50-100 cm
- 1-2 m
- 2-5 m
- >5 m

11. Si es una medusa que no es Cassiopea, proporcione el nombre del género o la especie. Algunas medusas Rhizostoma comunes se muestran en las imágenes de arriba.

____

12. Hasta donde usted sabe, ¿cuántas Cassiopea (u otras medusas Rhizostoma) había en un radio de 2 m de usted?

Mark only one oval.

- 0
- 1
- 2-5
- 5-10
- 10-20
- 20+

13. Proporcione una estimación del tamaño promedio de las medusas que estaban cerca de usted (en un radio de 2 m).

Mark only one oval.

- <5 cm
- 5-10 cm
- 10-15 cm
- 15-30 cm
- Other: ____________________

14. ¿Cuál fue la densidad más alta de Cassiopea (u otra medusa Rhizostoma) que vio en el área en la que estaba trabajando (individuos / metro cuadrado)?

____

15. ¿A qué distancia se encontraba de esta zona de mayor densidad de medusas?

Mark only one oval.

- <1 m
- 1-2 m
- 2-5 m
- 5-8 m
- >8 m
- No vi ninguna o no lo sé
- No había medusas Rhizostoma en la zona

16. ¿Hizo algo para apartar a las medusas? Esto incluye empujar, dar patadas en el agua circundante con aletas, pisar, etc.

Mark only one oval.

- Sí
- No
- Tal vez

17. ¿Cuánto tiempo se quedó en esta zona?

Mark only one oval.

- <5 min
- 5-10 min
- 10-15 min
- 15 -30 min
- 30-60 min
- 1 hr+

18. ¿Qué nivel de incomodidad experimentó? (1- cosquilleo leve, 3- quemadura e irritación, 5- dolor severo)

Mark only one oval.

	1	2	3	4	5	
Leve cosquilleo						Dolor severo

19. ¿Podría comparar esta incomodidad con alguna otra cosa?

20. ¿Tuvo alguna decoloración de la piel en este área después del malestar?

Mark only one oval.

- Sí
- No
- No se

21. ¿Hay algo más que le gustaría contarnos sobre esta experiencia?

22. ¿Tiene otra experiencia que quiera detallar?

Mark only one oval.

- Sí (hacer clic en sí le permitirá repetir estas preguntas para una experiencia adicional)
- No (si hace clic en no, accederá a los permisos de uso y al envío final) *Skip to question 58*

Experiencia 2

Por favor, describa aqui una experiencia.

23. ¿Dónde geográficamente (lo más específico posible) sintió “agua urticante”?

24. Si conoce las coordenadas de esta ubicación, inclúyalas a continuación; de lo contrario, omita esta pregunta.

____

25. Este lugar lo clasificaría como:

Mark only one oval.

- Manglar
- Laguna
- Presa
- Mar
- Acuario público (i.e. trabajo)
- Acuario privado (i.e. casa)
- Other: ____________________________________

26. Cuando sintió esto, ¿qué estaba haciendo (vadear, bucear, snorkel)?

Mark only one oval.

- Caminar por el agua
- Bucear
- Snorkel
- Trabajar en un acuario
- Other: ____________________________________

27. Si estaba cerca de una Cassiopea, ¿qué tan cerca estaba de la medusa?

Mark only one oval.

- <10 cm
- 10-50 cm
- 50-100 cm
- 1-2 m
- 2-5 m
- > 5 m
- No vi ninguna o no lo sé
- No estaba cerca de una Cassiopea

28. Igual que la pregunta anterior, pero para cualquier tipo de medusa. Proporcione las especies en la siguiente pregunta si es necesario.

Mark only one oval.

- <10 cm
- 10-50 cm
- 50-100 cm
- 1-2 m
- 2-5 m
- >5 m

29. Si es una medusa que no es Cassiopea, proporcione el nombre del género o la especie. Algunas medusas Rhizostoma comunes se muestran en las imágenes de arriba.

30. Hasta donde usted sabe, ¿cuántas Cassiopea (u otras medusas Rhizostoma) había en un radio de 2 m de usted?

Mark only one oval.

- 0
- 1
- 2-5
- 5-10
- 10-20
- 20+

31. Proporcione una estimación del tamaño promedio de las medusas que estaban cerca de usted (en un radio de 2 m).

Mark only one oval.

- <5 cm
- 5-10 cm
- 10-15 cm
- 15-30 cm
- Other: ______________________

32. ¿Cuál fue la densidad más alta de Cassiopea (u otra medusa Rhizostoma) que vio en el área en la que estaba trabajando (individuos / metro cuadrado)?

____

33. ¿A qué distancia se encontraba de esta zona de mayor densidad de medusas?

Mark only one oval.

- <1 m
- 1-2 m
- 2-5 m
- 5-8 m
- >8 m
- No vi ninguna o no lo sé
- No había medusas Rhizostoma en la zona

34. ¿Hizo algo para apartar a las medusas? Esto incluye empujar, dar patadas en el agua circundante con aletas, pisar, etc.

Mark only one oval.

- Sí
- No
- Tal vez

35. ¿Cuánto tiempo se quedó en esta zona?

Mark only one oval.

- <5 min
- 5-10 min
- 10-15 min
- 15 -30 min
- 30-60 min
- 1 hr+

36. ¿Qué nivel de incomodidad experimentó? (1- cosquilleo leve, 3- quemadura e irritación, 5- dolor severo)

Mark only one oval.

	1	2	3	4	5	
Leve cosquilleo	◯	◯	◯	◯	◯	Dolor severo

37. ¿Podría comparar esta incomodidad con alguna otra cosa?

____

38. ¿Tuvo alguna decoloración de la piel en este área después del malestar?

Mark only one oval.

- ◯ Sí
- ◯ No
- ◯ No se

39. ¿Hay algo más que le gustaría contarnos sobre esta experiencia?

____
____
____
____
____

40. ¿Tiene otra experiencia que quiera detallar?

Mark only one oval.

- Sí (hacer clic en sí le permitirá repetir estas preguntas para una experiencia adicional)
- No (si hace clic en no, accederá a los permisos de uso y al envío final) *Skip to question 58*

Experiencia 3

Por favor, describa aqui una experiencia.

41. ¿Dónde geográficamente (lo más específico posible) sintió "agua urticante"?

42. Si conoce las coordenadas de esta ubicación, inclúyalas a continuación; de lo contrario, omita esta pregunta.

43. Este lugar lo clasificaría como:

Mark only one oval.

- Manglar
- Laguna
- Presa
- Mar
- Acuario público (i.e. trabajo)
- Acuario privado (i.e. casa)
- Other: ______________________

44. Cuando sintió esto, ¿qué estaba haciendo (vadear, bucear, snorkel)?

Mark only one oval.

- Caminar por el agua
- Bucear
- Snorkel
- Trabajar en un acuario
- Other: ______________________

45. Si estaba cerca de una Cassiopea, ¿qué tan cerca estaba de la medusa?

Mark only one oval.

- <10 cm
- 10-50 cm
- 50-100 cm
- 1-2 m
- 2-5 m
- > 5 m
- No vi ninguna o no lo sé
- No estaba cerca de una Cassiopea

46. Igual que la pregunta anterior, pero para cualquier tipo de medusa. Proporcione las especies en la siguiente pregunta si es necesario.

Mark only one oval.

- <10 cm
- 10-50 cm
- 50-100 cm
- 1-2 m
- 2-5 m
- >5 m

47. Si es una medusa que no es Cassiopea, proporcione el nombre del género o la especie. Algunas medusas Rhizostoma comunes se muestran en las imágenes de arriba.

____

48. Hasta donde usted sabe, ¿cuántas Cassiopea (u otras medusas Rhizostoma) había en un radio de 2 m de usted?

Mark only one oval.

- 0
- 1
- 2-5
- 5-10
- 10-20
- 20+

49. Proporcione una estimación del tamaño promedio de las medusas que estaban cerca de usted (en un radio de 2 m).

Mark only one oval.

- <5 cm
- 5-10 cm
- 10-15 cm
- 15-30 cm
- Other: ____________________

50. ¿Cuál fue la densidad más alta de Cassiopea (u otra medusa Rhizostoma) que vio en el área en la que estaba trabajando (individuos / metro cuadrado)?

____

51. ¿A qué distancia se encontraba de esta zona de mayor densidad de medusas?

Mark only one oval.

- <1 m
- 1-2 m
- 2-5 m
- 5-8 m
- >8 m
- No vi ninguna o no lo sé
- No había medusas Rhizostoma en la zona

52. ¿Hizo algo para apartar a las medusas? Esto incluye empujar, dar patadas en el agua circundante con aletas, pisar, etc.

Mark only one oval.

- Sí
- No
- Tal vez

53. ¿Cuánto tiempo se quedó en esta zona?

Mark only one oval.

- <5 min
- 5-10 min
- 10-15 min
- 15 -30 min
- 30-60 min
- 1 hr+

54. ¿Qué nivel de incomodidad experimentó? (1- cosquilleo leve, 3- quemadura e irritación, 5- dolor severo)

Mark only one oval.

	1	2	3	4	5	
Leve cosquilleo	⬭	⬭	⬭	⬭	⬭	Dolor severo

55. ¿Podría comparar esta incomodidad con alguna otra cosa?

____

56. ¿Tuvo alguna decoloración de la piel en este área después del malestar?

Mark only one oval.

- ⬭ Sí
- ⬭ No
- ⬭ No se

57. ¿Hay algo más que le gustaría contarnos sobre esta experiencia?

____
____
____
____
____

Permisos de uso

Gracias por sus respuestas. Antes de enviar, seleccione la opción a continuación para divulgar esta información.

58. Permisos: ¿Acepta el uso de las respuestas de su encuesta en una comunicación de una revista pública sobre experiencias de picaduras de medusas sin contacto? *

Mark only one oval.

- No.
- Sí, puede usar mis respuestas como puntos de datos.
- Sí, puede usar mis respuestas como puntos de datos y mis respuestas anónimas escritas.
- Sí, puede usar mis respuestas como puntos de datos y mis respuestas escritas, por favor proporcione mi nombre por separado en los agradecimientos.

59. Nombre de la sección de agradecimientos: Gracias por sus respuestas.

This content is neither created nor endorsed by Google.

Google Forms
